# Supplementary material for: Nationally and regionally representative analysis of 1.65 million children aged under 5 years using a child-based human development index: A multi-country cross-sectional study
Source: PLoS Med. 2020 Mar 16;17(3):e1003054. doi: 10.1371/journal.pmed.1003054 (PMC7075547; doi:10.1371/journal.pmed.1003054)
Supplement: S1 File — (DOCX) [file pmed.1003054.s002.docx]

# S1 File: Study Design

Our study is a retrospective analysis of cross-sectional, multi-country data. The study is a non-interventional study and we do not use biocompatible, biomimetic or nature-based materials. Using exclusively secondary data, the study will not require randomization or blinding.

# Inclusion and exclusion criteria

*Inclusion criteria*

The study population will include all children born to eligible women in the DHS surveys. To assess child-based capabilities, we will limit the survey to households with children.

*Exclusion criteria*

We will exclude households without children or without data on our outcomes. Ethnicity, race, political orientation, religion and class do not constitute inclusion or exclusion criteria.

# Randomization

Since our secondary data sources are publicly available data, previously collected by the DHS, randomization is not required.

# Study termination criteria

Termination criteria include the withdrawal of permission from the DHS program.

# Statistical design

## Biometrical methods

Our analysis will proceed in the following steps. First, under-five survival rates at the individual or household level capture our measure of health. To increase the number of observations in each (administrative unit) cell, we will calculate under-five survival, maternal educational attainment, and household wealth by first- or second-level administrative unit available in each DHS. The administrative units considered include, e.g.: communes (DRC, Madagascar), counties (Kenya), departments (Colombia, Guatemala), districts (Bangladesh, Nepal, Pakistan, Uganda), governorates (Egypt), regions (Cambodia, Indonesia, Mozambique, Peru, Tanzania), provinces (Afghanistan, Turkey, Philippines), and states and union territories (India, Nigeria). The aggregate estimate for each component will be derived from individual-level data by averaging over individuals in a given subnational administrative unit ($\bar{Survival}$*_c,u_*, $\bar{Education}$*_c,u_* , and $\bar{Wealth}$*_c,u_*) where $\bar{Survival}$*_c,u_* represents the proportion of children in country-year *c* and administrative unit *u* who survived, and $\bar{Education}$*_c,u_* and $\bar{Wealth}$*_c,u_* represent the average maternal educational attainment and wealth quintile in that group. For most DHS surveys, averages at these subnational levels provide regionally representative estimates.

Second, for each country, we will graphically analyze under-five survival, education, and wealth. To do so, we will plot contour maps (akin to heat maps), where we will display *z* (under-five survival) as filled contours in (*y* = education, *x* = wealth). We will show three-dimensional data where under-five survival of the sample is represented by the color so that points with equal under-five survival in the graph will have the same color. For each *z* value of under-five survival, we will have a position for the two other *y* and *x* components of maternal education and wealth. To increase the resolution of these maps, we will set the number of levels in under-five survival to ten. For visualization purposes, we will normalize the range of each HDI component (rescaled from zero to 1) as follows:

Normalized Indicator = (Indicator - Range(min))/(Range(max) - Range(min)). (1)

To examine time trends in under-five survival by wealth and maternal educational attainment, we will also show results for selected countries with administrative boundaries that have largely remained consistent over the study period so that they are comparable over time. As an example, we will show the child-based HDI for the 36 states and Federal Capital Territory of Nigeria in 2003 and in 2013. This empirical approach will allow us to examine subnational shifts in health across the development spectrum and over time during the final run up towards the Millennium Development Goals.

Third, we will calculate a summary metric for the child-based capability index, as the geometric mean of the three normalized components (see Equation 2 below). While the geometric mean has been commonly used to summarize aggregate measured of human development, it has been applied infrequently to individual-level data from population-based surveys. We will calculate the child-based capability index at the national level for all countries *c* and administrative units *u*. When calculating the child-based capability index at the national level, we will similarly normalize the range of each component (Equation 1) and use data from the entire study population (as opposed to averaging across administrative units *u*). When calculating the child-based capability index at the regional level, we will also calculate the mean across administrative units *u* and the corresponding standard deviations (SD) of the child-based capability index to provide an estimate of within-country variation:

Capability Index_c,u_ = $\sqrt[3]{U5S_{normalized}*Edu_{normalized}*Wealth_{normalized}}$. (2)
