# Supplementary material for: Nationally and regionally representative analysis of 1.65 million children aged under 5 years using a child-based human development index: A multi-country cross-sectional study
Source: PLoS Med. 2020 Mar 16;17(3):e1003054. doi: 10.1371/journal.pmed.1003054 (PMC7075547; doi:10.1371/journal.pmed.1003054)
Supplement: S1 Text — (DOCX) [file pmed.1003054.s003.docx]

## **S1 Text. Measure of Household Wealth**

The DHS provides survey-specific wealth quintiles. Using country-specific measures of socioeconomic status, however, ignores absolute differences in wealth that can differ between countries (Fink et al., 2017; Poirier et al., 2018). We therefore constructed an asset index that allowed comparisons of the child-based capability index at the national level. To do so, we used an approach similar to the “International Wealth Index” (Smits & Steendijk, 2014). Specifically, we used data on 12 key housing characteristics and household ownership of 4 durable goods that were available across surveys to construct an international measure of household wealth.

Housing characteristics included the following: the availability of electricity; piped running water; a tube well or borehole; a dug or public well; a water spring; surface water (e.g., river, dam, lake, pond, stream, canal, irrigation channel); a water tanker truck or sachet water; a flush toilet; a pit latrine; a bucket toilet, composting toilet, or hanging toilet; a finished floor (e.g., polished wood, vinyl, asphalt strips, ceramic tiles, cement, carpet); a rudimentary floor (e.g., wood planks, palm, bamboo); and a natural floor (e.g., earth, sand, dung, dirt, mud). Household durable goods included: a radio, phone, television, and ownership of a motorized vehicle (e.g., motorbike, car).

For our main analysis, we extracted the first component in a principal component analysis (Filmer & Pritchett, 2001) and then divided this continuous household asset index into quintiles using the pooled sample. In sensitivity analyses, described in the main text and **S2 Text**, we examined alternative measures of household wealth.

**References:**

Fink G, Victora CG, Harttgen K, Vollmer S, Vidaletti LP, Barros AJD. Measuring Socioeconomic Inequalities With Predicted Absolute Incomes Rather Than Wealth Quintiles: A Comparative Assessment Using Child Stunting Data From National Surveys. *American Journal of Public Health*. 2017;107(4):550-5. doi: 10.2105/ajph.2017.303657.

Poirier MJP, Grignon M, Grépin KA, Dion ML. Transnational wealth-related health inequality measurement. *SSM - Population Health*. 2018;6:259-75. doi: 10.1016/j.ssmph.2018.10.009.

Smits J, Steendijk R. The International Wealth Index (IWI). *Social Indicators Research*. 2014;122(1):65-85. doi: 10.1007/s11205-014-0683-x.

Filmer D, Pritchett LH. Estimating Wealth Effects without Expenditure Data-or Tears: An Application to Educational Enrollments in States of India. *Demography*. 2001;38(1):115-32. doi: 10.2307/3088292.
