# Supplementary material for: Nationally and regionally representative analysis of 1.65 million children aged under 5 years using a child-based human development index: A multi-country cross-sectional study
Source: PLoS Med. 2020 Mar 16;17(3):e1003054. doi: 10.1371/journal.pmed.1003054 (PMC7075547; doi:10.1371/journal.pmed.1003054)
Supplement: S2 Text — (DOCX) [file pmed.1003054.s004.docx]

## **S2 Text. Additional Details on Sensitivity Analyses**

Alternative Goalposts

In our main approach to construct a national-level child-based capability index, we standardized the three outcomes to the minimum and maximum values across countries in our sample. These goalpost values act as ‘natural zeros’ and ‘aspirational goals’ from which components are standardized. As a sensitivity analysis, we generated a child-based capability index using alternative reference values building on the goalposts used to construct the HDI (UNDP, 2018).

We constructed three subindices as follows:

Education subindex (EI) = (Maternal_years_of_schooling – 0)/ (15 – 0) , (1)

where 15 years of maternal schooling is set as maximum in our data (15+); and EI = 1 if maternal schooling is 15, and EI = 0 if maternal schooling is 0;

Child health subindex (U5MI) = (Under_five_mortality – 0.003)/ (0.320 – 0.003), (2)

where 3 deaths per 1,000 is set as minimum in our data (< 3) (Japan, 2017); and U5MI = 0 if under-five mortality is 3, and U5MI = 1 if under-five mortality is 320 or more per 1,000 (Niger, 1991);

Wealth subindex (WI) = (Household_wealth_quintile – 1) / (5 – 1) , (3)

where 5 is set as maximum in our data; and WI = 1 if wealth quintile is 5, and WI = 0 if wealth quintile is 1.

We then re-constructed our summary metric for the child-based capability index at the national level using the three subindices described above (**S2** **Table**), rather than normalizing the range to the minimum and maximum values across all 55 countries in our sample.

Alternative Outcomes

As a sensitivity analysis, we used infant-survival (defined as survival within the first year of birth) instead of under-five survival. We also examined an alternative approach of calculating under-five child mortality. In our main approach, we created a binary variable indicating whether the child was alive or not at the time of the survey. As an alternative approach, however, we used synthetic cohort life tables (Croft et al., 2018). To do so, we combined mortality probabilities for small age segments based on real cohort mortality experience into the standard age segments. This approach allows full use of the most recent data and is specific for time periods.

In our main analysis, we constructed an international household asset index that allowed comparisons of the child-based capability index across countries (Poirier et al., 2018). This approach however makes the index somewhat more complex to implement by public health practitioners. We therefore present national-level results using the wealth index built into the DHS. The DHS wealth index takes into account country-specific differences and can be extended in a straightforward manner to other settings. We also considered using predicted absolute incomes to measure household wealth by simulating household income from an asset index and macroeconomic data (Fink et al., 2017), but which would have relied on additional assumptions.

Comparison with other Indices

We show side-by-side comparisons with other commonly used indices at both the national and sub-national level. At the national level, we compared our child-based capability index with the United Nations Development Programme’s Human Development Index (HDI), the World Bank’s Human Capital Index (HCI), and the Socio-Demographic Index (SDI) developed by the Global Burden of Disease study. We show index values and rankings for each to allow direct comparisons of differences in ranking, as well as differences in the absolute values and range of the index. We find that the correlation of our child-based capability index and these commonly used indices at the national level was highest for the SDI (*r =* 0.96, *p*-value for test of *r* = 0 < 0.01) and lowest for the HCI (*r =* 0.84, *p*-value for test of *r* = 0 < 0.01) (**S3** **Table**).

At the sub-national level, we also show direct comparisons with the household-based index developed by Harttgen and Klasen (2012). To do so, we have replicated the household-based index developed by Harttgen and Klasen while taking the case of Zimbabwe DHS survey (2015) as an example. Our direct comparison of the two methods reveals two findings: i) we find differences in the rank of regions (the region Manicaland, for instance, performs worse on the child-based index, compared to the index developed by Harttgen and Klasen [difference in rank position: -2]; and ii) our child-based index has a wider range (0.445 – 0.766, **S4** **Table**), suggesting that the child-based index may pick up relatively larger within-country differences.

**References:**

Croft T, Aileen M, Courtney K. Guide to DHS Statistics. Rockville, Maryland, USA: ICF, 2018.

Fink G, Victora CG, Harttgen K, Vollmer S, Vidaletti LP, Barros AJD. Measuring Socioeconomic Inequalities With Predicted Absolute Incomes Rather Than Wealth Quintiles: A Comparative Assessment Using Child Stunting Data From National Surveys. *American Journal of Public Health*. 2017;107(4):550-5. doi: 10.2105/ajph.2017.303657.

Global Burden of Disease Collaborative Network. Global Burden of Disease Study 2017 (GBD 2017) Socio-Demographic Index (SDI) 1950–2017. In: Institute for Health Metrics and Evaluation (IHME), editor. Seattle, United States 2018.

Harttgen K, Klasen S. A Household-Based Human Development Index. *World Development*. 2012;40(5):878-99. doi: 10.1016/j.worlddev.2011.09.011.

Kraay A. Methodology for a World Bank Human Capital Index (English). Washington, D.C.: World Bank Group, 2018.

Poirier MJP, Grignon M, Grépin KA, Dion ML. Transnational wealth-related health inequality measurement. *SSM - Population Health*. 2018;6:259-75. doi: 10.1016/j.ssmph.2018.10.009.

United Nations Development Programme (UNDP). Human development indices and indicators: 2018 statistical update. 2018.
