# Supplementary material for: Nationally and regionally representative analysis of 1.65 million children aged under 5 years using a child-based human development index: A multi-country cross-sectional study
Source: PLoS Med. 2020 Mar 16;17(3):e1003054. doi: 10.1371/journal.pmed.1003054 (PMC7075547; doi:10.1371/journal.pmed.1003054)
Supplement: S1 Fig — (DOCX) [file pmed.1003054.s005.docx]

## S1 Fig. Underlying Principles of the Child-based Capability Index


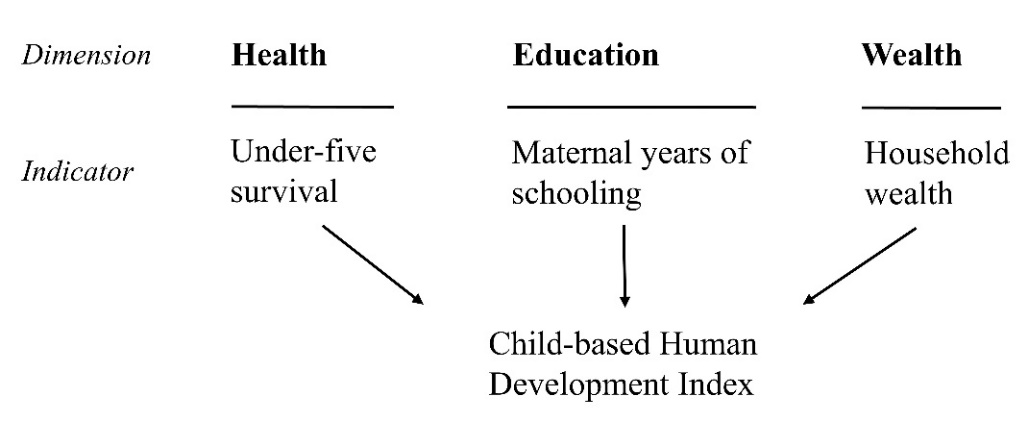


*Notes:* Authors’ conceptual framework for the child-based capability index which, building on the human development index, incorporates three dimensions: health (under-five survival); education (maternal years of schooling); and wealth (household wealth). All three indicators are calculated based on individual-level data from nationally and regionally representative population-based surveys.
