# Supplementary figures and images for: Nationally and regionally representative analysis of 1.65 million children aged under 5 years using a child-based human development index: A multi-country cross-sectional study
Source: PLoS Med. 2020 Mar 16;17(3):e1003054. doi: 10.1371/journal.pmed.1003054 (PMC7075547; doi:10.1371/journal.pmed.1003054)

## S2 Fig. Child-based Capability Index using Infant Mortality


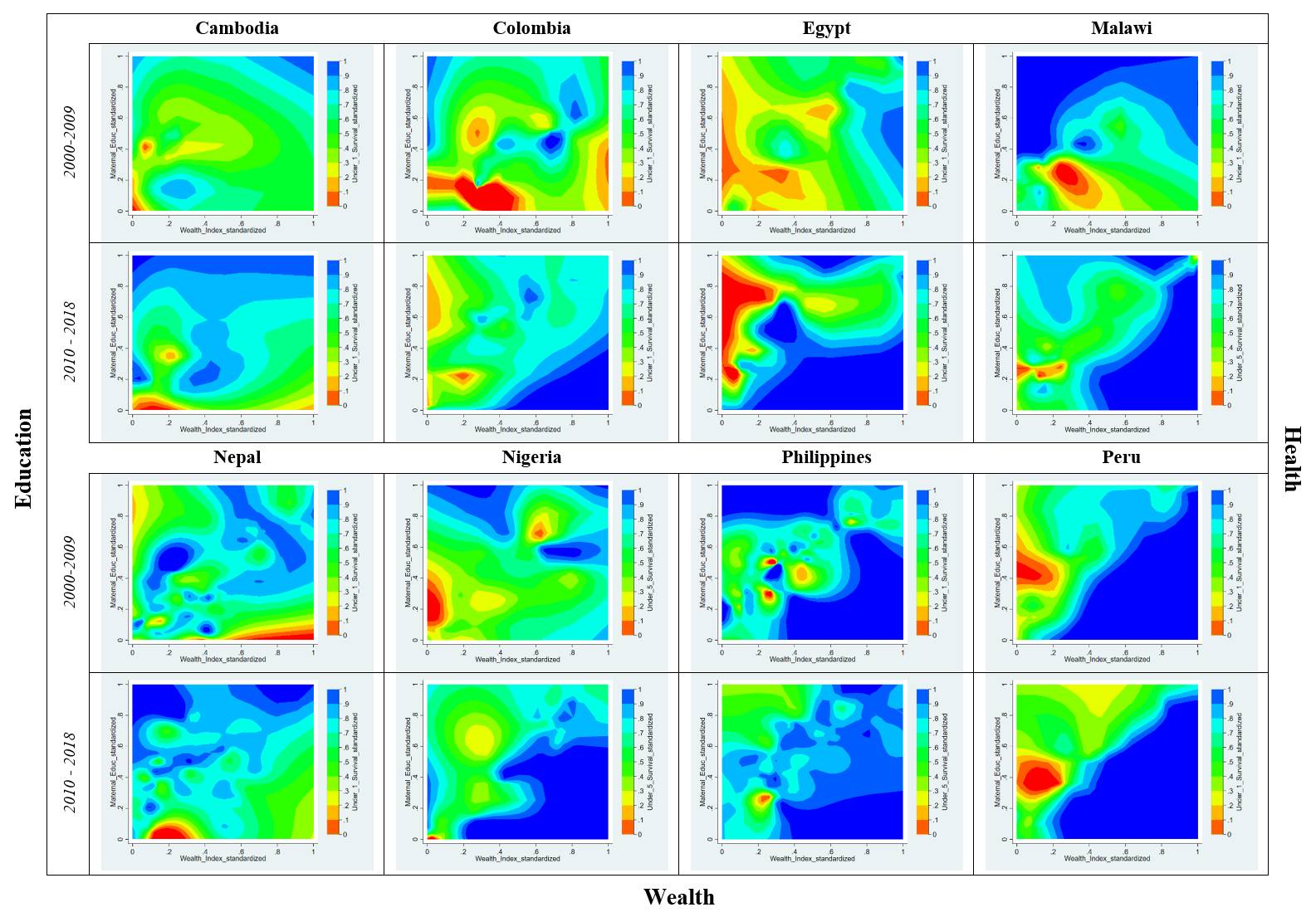

Supplement: S2 Fig — (DOCX) [file pmed.1003054.s006.docx]

## S3 Fig. Child-based Capability Index using the Full Birth History of Women in the DHS


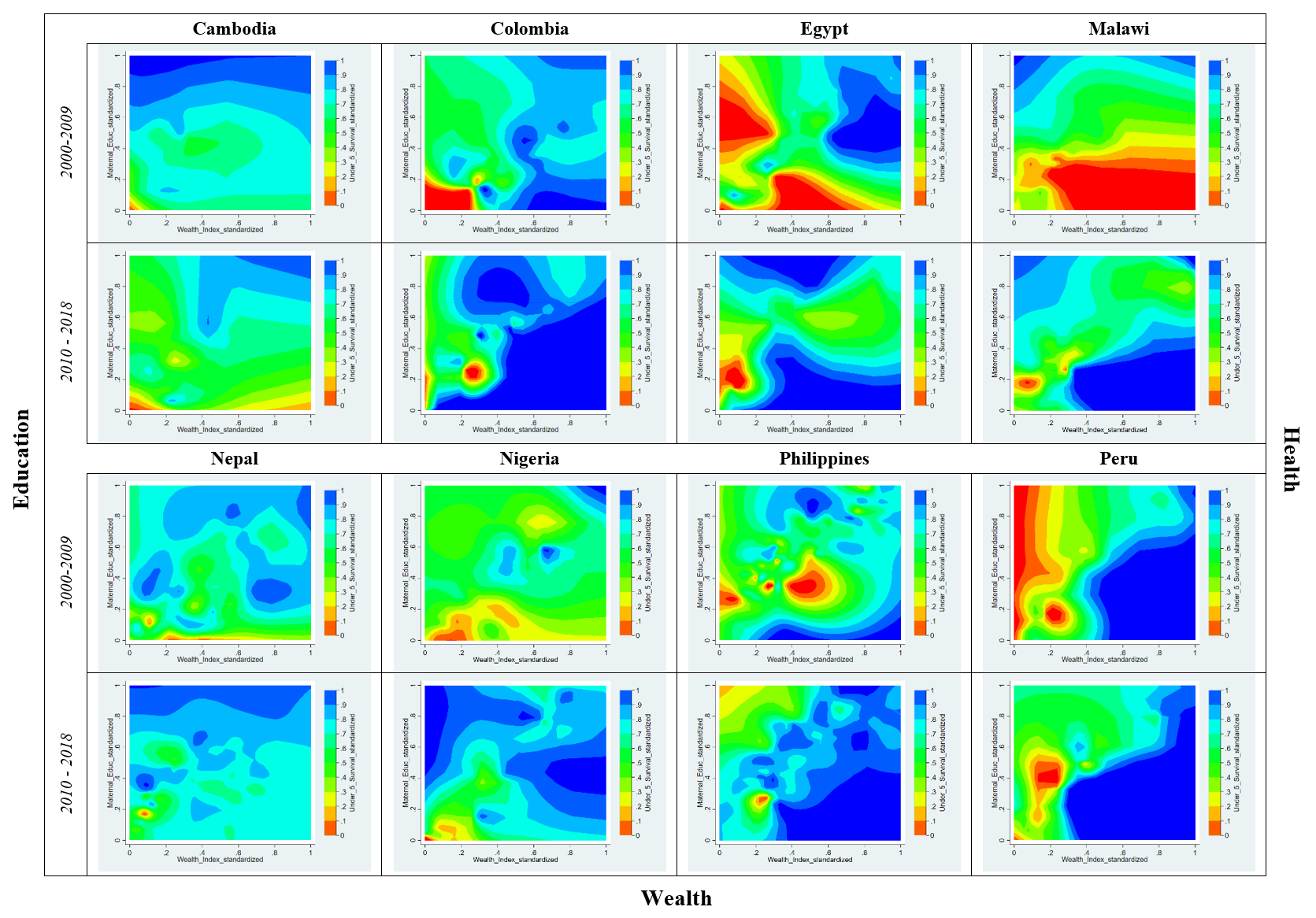

Supplement: S3 Fig — (DOCX) [file pmed.1003054.s007.docx]
