# Supplementary material for: Nationally and regionally representative analysis of 1.65 million children aged under 5 years using a child-based human development index: A multi-country cross-sectional study
Source: PLoS Med. 2020 Mar 16;17(3):e1003054. doi: 10.1371/journal.pmed.1003054 (PMC7075547; doi:10.1371/journal.pmed.1003054)
Supplement: S4 Fig — (DOCX) [file pmed.1003054.s008.docx]

## S4 Fig. Heterogeneity in Child-based Capabilities, by Sex


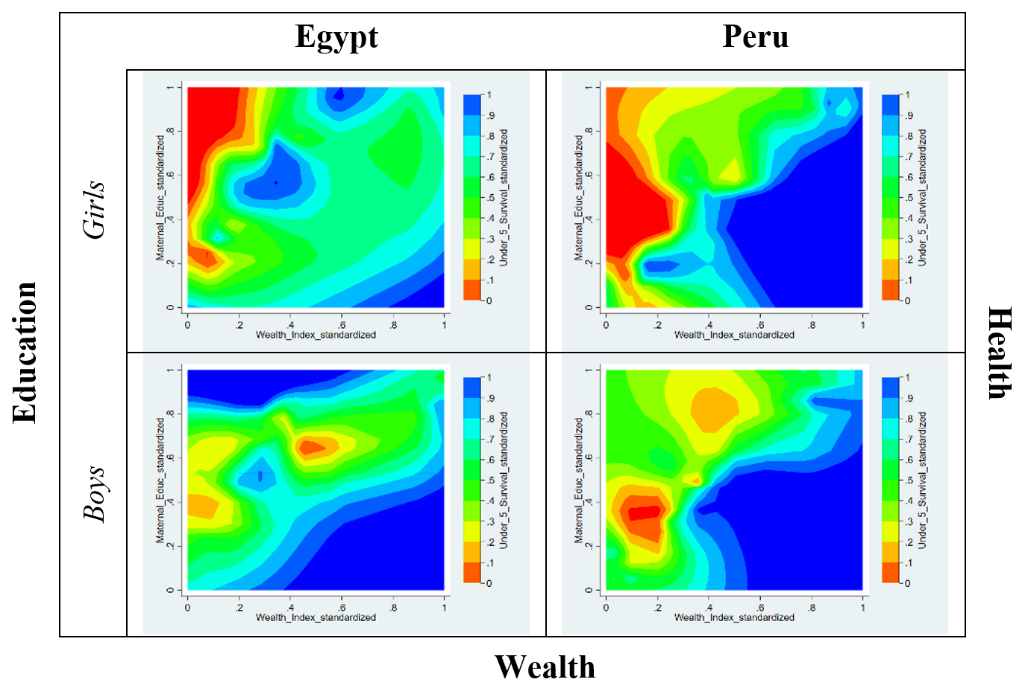


*Notes:* Figure shows three axes, including health (under-five survival), wealth (household wealth), and education (maternal education), using the most recently available DHS survey for Egypt and Peru, by a child’s sex.
