# Supplementary material for: Nationally and regionally representative analysis of 1.65 million children aged under 5 years using a child-based human development index: A multi-country cross-sectional study
Source: PLoS Med. 2020 Mar 16;17(3):e1003054. doi: 10.1371/journal.pmed.1003054 (PMC7075547; doi:10.1371/journal.pmed.1003054)
Supplement: S1 Table — (DOCX) [file pmed.1003054.s009.docx]

S1 Table. Child-based Capability Index by Subnational Region

[Child-based Capability Index by Region in Afghanistan, 2015-16 3](#_Toc33561306)

[Child-based Capability Index by Region in Albania, 2017-18 4](#_Toc33561307)

[Child-based Capability Index by Region in Angola, 2015-16 5](#_Toc33561308)

[Child-based Capability Index by Region in Armenia, 2015-16 6](#_Toc33561309)

[Child-based Capability Index by Region in Bangladesh, 2014 7](#_Toc33561310)

[Child-based Capability Index by Region in Benin, 2017-18 8](#_Toc33561311)

[Child-based Capability Index by Region in Burkina Faso, 2010 9](#_Toc33561312)

[Child-based Capability Index by Region in Burundi, 2016-17 10](#_Toc33561313)

[Child-based Capability Index by Region in Cambodia, 2014 11](#_Toc33561314)

[Child-based Capability Index by Region in Cameroon, 2011 12](#_Toc33561315)

[Child-based Capability Index by Region in Chad, 2014-15 13](#_Toc33561316)

[Child-based Capability Index by Region in Colombia, 2015 14](#_Toc33561317)

[Child-based Capability Index by Region in Comoros, 2012 15](#_Toc33561318)

[Child-based Capability Index by Region in Congo, Dem. Rep., 2013-14 16](#_Toc33561319)

[Child-based Capability Index by Region in Congo, Rep., 2011-12 17](#_Toc33561320)

[Child-based Capability Index by Region in Cote d’Ivoire, 2011-12 18](#_Toc33561321)

[Child-based Capability Index by Region in Dominican Republic, 2013 19](#_Toc33561322)

[Child-based Capability Index by Region in Egypt, Arab Rep., 2014 20](#_Toc33561323)

[Child-based Capability Index by Region in Ethiopia, 2016 21](#_Toc33561324)

[Child-based Capability Index by Region in Gabon, 2012 22](#_Toc33561325)

[Child-based Capability Index by Region in The Gambia, 2013 23](#_Toc33561326)

[Child-based Capability Index by Region in Ghana, 2014 24](#_Toc33561327)

[Child-based Capability Index by Region in Guatemala, 2014-15 25](#_Toc33561328)

[Child-based Capability Index by Region in Guinea, 2012 26](#_Toc33561329)

[Child-based Capability Index by Region in Haiti, 2016-17 27](#_Toc33561330)

[Child-based Capability Index by Region in Honduras, 2011-12 28](#_Toc33561331)

[Child-based Capability Index by Region in India, 2015-16 29](#_Toc33561332)

[Child-based Capability Index by Region in Indonesia, 2012 30](#_Toc33561333)

[Child-based Capability Index by Region in Jordan, 2012 31](#_Toc33561334)

[Child-based Capability Index by Region in Kenya, 2014 32](#_Toc33561335)

[Child-based Capability Index by Region in Kyrgyz Republic, 2012 33](#_Toc33561336)

[Child-based Capability Index by Region in Lesotho, 2014 34](#_Toc33561337)

[Child-based Capability Index by Region in Liberia, 2013 35](#_Toc33561338)

[Child-based Capability Index by Region in Malawi, 2015-16 36](#_Toc33561339)

[Child-based Capability Index by Region in Maldives, 2016-17 37](#_Toc33561340)

[Child-based Capability Index by Region in Mali, 2012-13 38](#_Toc33561341)

[Child-based Capability Index by Region in Mozambique, 2011 39](#_Toc33561342)

[Child-based Capability Index by Region in Namibia, 2013 40](#_Toc33561343)

[Child-based Capability Index by Region in Nepal, 2016 41](#_Toc33561344)

[Child-based Capability Index by Region in Niger, 2012 42](#_Toc33561345)

[Child-based Capability Index by Region in Nigeria, 2013 43](#_Toc33561346)

[Child-based Capability Index by Region in Pakistan, 2017-18 44](#_Toc33561347)

[Child-based Capability Index by Region in Peru, 2012 45](#_Toc33561348)

[Child-based Capability Index by Region in Philippines, 2017 46](#_Toc33561349)

[Child-based Capability Index by Region in Rwanda, 2014-15 47](#_Toc33561350)

[Child-based Capability Index by Region in Senegal, 2017 48](#_Toc33561351)

[Child-based Capability Index by Region in Sierra Leone, 2013 49](#_Toc33561352)

[Child-based Capability Index by Region in South Africa, 2016 50](#_Toc33561353)

[Child-based Capability Index by Region in Tajikistan, 2017 51](#_Toc33561354)

[Child-based Capability Index by Region in Tanzania, 2015-16 52](#_Toc33561355)

[Child-based Capability Index by Region in Timor-Leste, 2016 53](#_Toc33561356)

[Child-based Capability Index by Region in Togo, 2013-14 54](#_Toc33561357)

[Child-based Capability Index by Region in Uganda, 2016 55](#_Toc33561358)

[Child-based Capability Index by Region in Zambia, 2013-14 56](#_Toc33561359)

[Child-based Capability Index by Region in Zimbabwe, 2015 57](#_Toc33561360)

## Child-based Capability Index by Region in Afghanistan, 2015-16

## Child-based Capability Index by Region in Albania, 2017-18

## Child-based Capability Index by Region in Angola, 2015-16

## Child-based Capability Index by Region in Armenia, 2015-16

## Child-based Capability Index by Region in Bangladesh, 2014

## Child-based Capability Index by Region in Benin, 2017-18

## Child-based Capability Index by Region in Burkina Faso, 2010

## Child-based Capability Index by Region in Burundi, 2016-17

## Child-based Capability Index by Region in Cambodia, 2014

## Child-based Capability Index by Region in Cameroon, 2011

## Child-based Capability Index by Region in Chad, 2014-15

## Child-based Capability Index by Region in Colombia, 2015

## Child-based Capability Index by Region in Comoros, 2012

## Child-based Capability Index by Region in Congo, Dem. Rep., 2013-14

## Child-based Capability Index by Region in Congo, Rep., 2011-12

## Child-based Capability Index by Region in Cote d’Ivoire, 2011-12

## Child-based Capability Index by Region in Dominican Republic, 2013

## Child-based Capability Index by Region in Egypt, Arab Rep., 2014

## Child-based Capability Index by Region in Ethiopia, 2016

## Child-based Capability Index by Region in Gabon, 2012

## Child-based Capability Index by Region in The Gambia, 2013

## Child-based Capability Index by Region in Ghana, 2014

## Child-based Capability Index by Region in Guatemala, 2014-15

## Child-based Capability Index by Region in Guinea, 2012

## Child-based Capability Index by Region in Haiti, 2016-17

## Child-based Capability Index by Region in Honduras, 2011-12

## Child-based Capability Index by Region in India, 2015-16

## Child-based Capability Index by Region in Indonesia, 2012

## Child-based Capability Index by Region in Jordan, 2012

## Child-based Capability Index by Region in Kenya, 2014

## Child-based Capability Index by Region in Kyrgyz Republic, 2012

## Child-based Capability Index by Region in Lesotho, 2014

## Child-based Capability Index by Region in Liberia, 2013

## Child-based Capability Index by Region in Malawi, 2015-16

## Child-based Capability Index by Region in Maldives, 2016-17

## Child-based Capability Index by Region in Mali, 2012-13

## Child-based Capability Index by Region in Mozambique, 2011

## Child-based Capability Index by Region in Namibia, 2013

## Child-based Capability Index by Region in Nepal, 2016

## Child-based Capability Index by Region in Niger, 2012

## Child-based Capability Index by Region in Nigeria, 2013

## Child-based Capability Index by Region in Pakistan, 2017-18

## Child-based Capability Index by Region in Peru, 2012

## Child-based Capability Index by Region in Philippines, 2017

## Child-based Capability Index by Region in Rwanda, 2014-15

## Child-based Capability Index by Region in Senegal, 2017

## Child-based Capability Index by Region in Sierra Leone, 2013

## Child-based Capability Index by Region in South Africa, 2016

## Child-based Capability Index by Region in Tajikistan, 2017

## Child-based Capability Index by Region in Tanzania, 2015-16

## Child-based Capability Index by Region in Timor-Leste, 2016

## Child-based Capability Index by Region in Togo, 2013-14

## Child-based Capability Index by Region in Uganda, 2016

## Child-based Capability Index by Region in Zambia, 2013-14

## Child-based Capability Index by Region in Zimbabwe, 2015
