# Supplementary material for: Nationally and regionally representative analysis of 1.65 million children aged under 5 years using a child-based human development index: A multi-country cross-sectional study
Source: PLoS Med. 2020 Mar 16;17(3):e1003054. doi: 10.1371/journal.pmed.1003054 (PMC7075547; doi:10.1371/journal.pmed.1003054)
Supplement: S2 Table — (DOCX) [file pmed.1003054.s010.docx]

## S2 Table. Child-based Capability Index using Alternative Specifications

*Notes:* Table shows the child-based capability index for each country. The child-based capability index was calculated using the geometric mean of under-five survival (1 *minus* under-five mortality), maternal schooling (years), and household wealth (quintiles), each at the national level, and separately by country. In column 5, we calculated the child-based capability index using the arithmetic mean (as opposed to the geometric mean). In column 7, each of the three components was normalized to a subindex representing alternative ‘goalpost’ (reference) values. In column 9, we calculated the child-based capability index using the survey-specific wealth index built into the DHS (instead of calculating our own international asset index which allowed comparisons across countries).
