# Supplementary material for: Nationally and regionally representative analysis of 1.65 million children aged under 5 years using a child-based human development index: A multi-country cross-sectional study
Source: PLoS Med. 2020 Mar 16;17(3):e1003054. doi: 10.1371/journal.pmed.1003054 (PMC7075547; doi:10.1371/journal.pmed.1003054)
Supplement: S3 Table — (DOCX) [file pmed.1003054.s011.docx]

## S3 Table. Comparison with other National-level Indices

*Notes*: Table shows the child-based capability index at the national level in comparison to other commonly used national-level indices, including the Human Development Index (HDI), Human Capital Index (HCI), and Socio-Demographic Index (SDI). The HDI and SDI were available for all countries and survey years; whereas the HCI was not available prior to 2017 and not available for the Maldives for any year. IHME: Institute of Health Metrics and Evaluation. UNDP: United Nations Development Programme. n/a: not available.
