# Supplementary material for: Nationally and regionally representative analysis of 1.65 million children aged under 5 years using a child-based human development index: A multi-country cross-sectional study
Source: PLoS Med. 2020 Mar 16;17(3):e1003054. doi: 10.1371/journal.pmed.1003054 (PMC7075547; doi:10.1371/journal.pmed.1003054)
Supplement: S4 Table — (DOCX) [file pmed.1003054.s012.docx]

## S4 Table. Comparison with other sub-National Index

*Notes:* Table compares the child-based human development index with the household-based human development index developed by Harttgen and Klasen (2012). Source: authors’ calculations using data from the Demographic and Health Survey for Zimbabwe (2015).
