# Supplementary material for: Nationally and regionally representative analysis of 1.65 million children aged under 5 years using a child-based human development index: A multi-country cross-sectional study
Source: PLoS Med. 2020 Mar 16;17(3):e1003054. doi: 10.1371/journal.pmed.1003054 (PMC7075547; doi:10.1371/journal.pmed.1003054)
Supplement: Attachment — Submitted filename: HDI Response 2 Dec 2019.docx [file pmed.1003054.s013.docx]

**Editors’ comments**

*In the data statement, please make that "Data are ...". We suggest adding "... and all relevant study data are included in the paper" or similar.*

**Authors’ response:**

We have now edited the data statement accordingly:

“Data are available from the DHS Program (<https://dhsprogram.com/>) and all relevant study data are included in the paper and its supporting files.”

*Please restructure your title so that the portion after the colon consists only of the study descriptor, e.g., "...: a cross-sectional study". We suggest: "Nationally and regionally representative analysis of 1.65 million children aged under 5 years using a child-based human development index: a cross-sectional study".*

**Authors’ response:**

We have now restructured the title (noting that we used 1.66 to round the total number of 1,657,272 children). However, we would be happy to change this to 1.65 at the behest of the editors.

“Nationally and regionally representative analysis of 1.66 million children aged under 5 years using a child-based human development index: a cross-sectional study”

*Please combine the "methods" and "findings" subsections of your abstract, and add a new final sentence to the combined subsection to summarize the study's main limitations.*

**Authors’ response:**

We have combined the methods and findings subsections of the abstract, and added a new final sentence to the combined subsection which summarizes the study’s main limitations:

“**Methods and Findings:** We use nationally and regionally representative individual-level data from Demographic and Health Surveys for 55 low- and middle-income countries to examine patterns in human capabilities at the national and regional levels, between 2000 and 2017 (*N*=1,657,272 children under age 5). We graphically analyze human capabilities, separately for each country, and propose a novel child-based Human Development Index (HDI), based on under-five survival, maternal educational attainment, and measures of a child’s household wealth.

We find considerable heterogeneity in child health across countries as well as within countries. At the national level, the child-based HDI ranged from 0.140 in Niger (with mean across first-level administrative units = 0.257 and standard deviation [SD]: 0.106) to 0.755 in Albania (with mean across first-level administrative units = 0.569 and SD: 0.084). There are improvements over time overall, between the 2000s and 2010s, although this is not the case for all countries included in our study. In Colombia, Egypt, and Nigeria, for instance, under-five survival improved over time at most levels of maternal education and wealth. In contrast, in the Philippines, we found little improvement in under-five survival across the development spectrum and over time. In these countries, the persistent location of geographical areas of poor child health across both the development spectrum and time may indicate within-country poverty traps.

Limitations of our study included its descriptive nature, lack of information beyond first- and second-level administrative units, and limited generalizability beyond the countries analyzed.” (Abstract, revised manuscript)

*After the abstract, we ask you to add a new and accessible "author summary" section in non-identical prose. You may find it helpful to consult one or two recent research papers published in PLOS Medicine to get a sense of the preferred style.*

**Authors’ response:**

We have now added an author summary on page 3 of the revised manuscript.

Why was this study done?

- Education and health are both constituents of human capital that enable people to earn higher wages and enhance people’s capabilities to lead fulfilling lives.
- A better understanding of how these different human capabilities are produced together could point to opportunities to help jointly reduce the wide disparities in health and education across populations.
- Few recent efforts, however, have been made to expand measurement to include education, health, as well as economic growth – and, to our knowledge, none has looked at indicators that are specifically focused on improving child health.

What did the researchers do and find?

- We use nationally and regionally representative individual-level data from 1.66 million under-five children in 55 low- and middle-income countries to examine patterns in human capabilities at the national and regional levels, between 2000 and 2017.
- We graphically analyze human capabilities, separately for each country, and propose a novel child-based Human Development Index, based on under-five survival, maternal educational attainment, and measures of a child’s household wealth.
- We find considerable heterogeneity in child-based capabilities across countries –ranging from 0.140 in Niger to 0.755 in Albania – as well as within countries.
- We find improvements over time overall although this is not the case for all countries. The persistent location of geographical areas of poor child health across both the development spectrum and time may indicate within-country poverty traps.

What do these findings mean?

- Areas of chronic deprivation across and within countries may require alternative policy approaches to improving child capabilities in low resource settings.
- These findings point decision-makers to more targeted efforts to further reduce persistent health disparities toward the Sustainable Development Goals.
- Our approach to compute a capability index is straightforward to implement and could be easily adapted to other indicators, populations, and settings.

*Please trim the paragraph at lines 84-96 to briefly convey the aims of your study, removing mentions of your findings ("we identify ...") and moving elements of discussion to the discussion section.*

**Authors’ response:**

We have now trimmed the paragraph (now at lines 87-96), as requested, removing mentions of our findings and elements of discussion to the Discussion section to more concisely describe the study aims:

“In this study, we aim to make two contributions to the literature on human development and capabilities, while taking the illustrative example of under-five mortality. First, we use nationally and regionally representative individual-level data from 55 LMICs to show current patterns as well as trends in human capabilities at the national and regional levels. We show heat maps of health, education, and wealth, and we identify major areas of high under-five mortality stratified by wealth and parental educational attainment levels. Second, we use under-five mortality, household wealth, and maternal educational attainment at the sub-national level as our measures of health, wealth, and education, respectively. Our approach to compute a child-based capability index is straightforward to implement as a summary metric for decision-makers seeking to bolster human capabilities in the post-2015 development era, and could be easily adapted to other indicators.” (p.5, revised manuscript, Introduction)

*Early in the methods section of your main text, please state whether the study had a protocol or prespecified analysis plan, and if so attach the relevant document(s) as a supplementary file (referred to in the methods section). Please highlight analyses that were not prespecified.*

**Authors’ response:**

This study was pre-registered with the Heidelberg University Hospital Ethics Committee (S-271/2019). We have now stated this in the Methods section and have added the relevant document.

“This study was pre-registered and approved by the Heidelberg University Hospital Ethics Committee (S-271/2019) (File S1).” (p.12, revised manuscript, Methods Section)

*We ask you to restructure the first paragraph of your discussion section so that it consists predominantly of a summary of the study's findings. Aspects of discussion can appear in subsequent paragraphs.*

**Authors’ response:**

We thank the editors for this excellent suggestion. We have now restructured the Discussion section so that the first paragraph consists predominantly of a summary of the findings.

“Using nationally and regionally representative data from 1.66 million children, this retrospective analysis makes a number of contributions to our understanding of where human capabilities are produced jointly [27]. First, we find substantial heterogeneity in child health across countries as well as within countries and over time. In particular, geographical areas of low under-five survival within countries existed in expected areas – i.e., areas with relatively low levels of average maternal educational attainment and household wealth – as well as in unexpected areas along the development spectrum (displayed as red zones in Figure 1). Second, our study shows trends in child health over time. We find improvements over time overall between 2000 and 2017, although this is not the case for all countries included in our study. In Colombia, Egypt, and Nigeria, for instance, under-five survival improved in geographical areas at most levels of average maternal educational attainment and household wealth. Third, our analysis reveals changes in the location of areas of low under-five survival both along the development spectrum within countries and over time. In Egypt and Malawi, for instance, low under-five survival shifted from areas with on average wealthier households to areas with on average poorer households. In contrast, in the Philippines, the consistent location of areas of poor child health across both the development spectrum and over time may indicate areas of chronic deprivation among populations at risk. These areas may indicate within-country poverty traps and require alternative policy approaches to improving child health.

While child health was generally better in wealthier areas, we identified a number of areas of poor child health with relatively high average levels of human capital. In Egypt and Peru, for instance, low under-five survival was observed in areas with high levels of educational attainment (upper left corner in Figure 1), in particular for girls under-five (see Figure S4 for results disaggregated by a child’s sex in Egypt and Peru) [28]. Although this finding appears counterintuitive, a growing literature suggests mixed child health returns to additional years of maternal schooling [29-32]. A recent systematic review examined evidence for a causal link between maternal education and child health, and found that parental schooling may play a more muted role in parents’ decisions about whether and how much to invest in their children’s health than previously suggested [33]. Moreover, even well-educated parents seeking to correct common health risks in their children may lack access to high quality primary healthcare services or face high out-of-pocket expenditures [34]. There may also be threshold levels to see an effect of parental schooling on child health outcomes (e.g., primary schooling alone may not be enough to see a protective effect on child health). One reason for threshold levels may be overcrowding or poor quality of instruction at lower school levels [35].” (p.21, Discussion section, revised manuscript)

*Throughout the text, please use the past tense consistently to describe analyses, e.g., at line 85 "... we used nationally and regionally ...".*

**Authors’ response:**

We thank the editor for bringing this to our attention. We have now edited the paper to consistently use past tense to describe analyses throughout the text – see, e.g.:

“First, we used nationally and regionally representative individual-level data from 55 LMICs to show current patterns as well as trends in human capabilities at the national and regional levels.” (p.5, Introduction section, revised manuscript)

“Second, we used under-five mortality, household wealth, and maternal educational attainment at the sub-national level as our measures of health, wealth, and education, respectively.” (p.5, Introduction section, revised manuscript)

*Please substitute 1.65 million for 1.7 million throughout.*

**Authors’ response:**

We have now changed 1.7 million to 1.66 million (noting that we used 1.66 to round the total number of 1,657,272 children). However, we would be happy to change this to 1.65 at the behest of the editors.

“Using nationally and regionally representative data from 1.66 million children, this retrospective analysis makes a number of contributions to our understanding of where human capabilities are produced jointly [27].” (p.21, revised manuscript, Discussion section)

*Please consider possible stigmatizing interpretations of the term "hot spots".*

**Authors’ response:**

We thank the editors for bringing this to our attention. We have now entirely removed the term from the paper, and have rephrased the corresponding sentences using more appropriate, neutral terms.

*In your reference list, please abbreviate journal names as appropriate.*

**Authors’ response:**

We have now abbreviated all journal names in accordance with author guidelines.

*Please add a completed checklist for the most appropriate reporting guideline, which may be RECORD, as a supplementary file, referred to in your methods section. In the checklist, individual items should be referred to by section (e.g., "Methods") and paragraph number rather than by line or page numbers, as the latter generally change in the event of publication.*

**Authors’ response:**

We have now added a completed RECORD checklist as a supplementary file.

“RECORD checklist” (Checklist, revised manuscript)

Reference:

Benchimol EI, Smeeth L, Guttmann A, Harron K, Moher D, Petersen I, Sørensen HT, von Elm E, Langan SM; RECORD Working Committee. The REporting of studies Conducted using Observational Routinely-collected health Data (RECORD) statement. *PLoS Med*. 2015 Oct 6;12(10):e1001885.

**Reviewers’ comments**

*Reviewer #1:*

*The paper presents an interesting method of triangulating maternal education and household wealth with a third factor that is health-related, in this case, under-5 child survival. The purposes are to 1) create a new child-based human development index (HDI) at the sub-national (e.g., province) and national levels; and 2) create visual representations in the form of heat maps that show where the highest child mortality is located along regional education and wealth dimensions. The authors provide a sample of countries with data from 2 different decades to show evidence of change (or lack of change) over time using the heat maps.*

**Authors’ response:**

We thank the reviewer for these comments.

*I think that the greatest value of the author's approach is developing an index that operates at the sub-national level, which can inform country policy makers and program implementers of at-risk regions within the country. At the national level, I'm unconvinced that the child-based HDI will reveal important insights that could not be obtained from ranking or comparing countries using a number of other indices (e.g., the WB's Human Capital Index, the SDI, WPS, or others). Can the authors provide evidence of improved sensitivity to inequalities or needs with their index compared to some of the other popular indices (not just the HDI)? In other words, what would motivate a researcher to choose their index at the national level over others?*

**Authors’ response:**

A key strength of our approach lies in developing an index that operates at the sub-national level, as noted by the reviewer. At the national level, however, there are a number of notable advantages. First, we calculated the index at the national level as the geometric mean of the three components of the index. Other national-level indices, such as the “Child Development Index”, which focuses on health and nutrition, calculate an index using the arithmetic mean, so that each have equal weighting in the combined index score. This method of aggregation, however, allows a higher score in one component to ‘substitute’ for lower values for the other components. In contrast, by using the geometric mean, low achievement in one component cannot be linearly compensated for by high achievement in another component.

Second, our child-based index can be disaggregated along other socio-demographic dimensions at the national level in a straightforward manner by researchers and public health practitioners, in addition to geographical dimensions within countries. By doing so, our approach may pick up heterogeneity along socio-demographic dimensions at the national level (e.g., by sex, migration status) that may otherwise be masked at the level of the full population. Bearing in mind the reviewer’s comments, we have now illustrated such an analysis in our paper (shown in Figure S4 for results by sex).

Third, our approach can quantify inequalities at the national level. We show results for the national-level mean index and corresponding standard deviations (SD) to provide estimates of variation (Table 2).

References:

Cobham A, Molina N, Garde M (2012). The Child Development Index 2012. London, UK: The Save the Children Fund.

*I have several requests for additional clarification regarding the indices: 1) The DHS supplies survey sampling weights to recreate a representative sample of the population. Were these used when calculating the indices? If so, please add this information. If not, shouldn't they be?*

**Authors’ response:**

We used the DHS-provided sample weights in all our analyses, which is the recommended strategy by the reviewer. We have now further clarified these points in the Methods section.

“Survey sample weights were used as provided by the DHS.” (notes, Table 1)

“We used DHS survey sample weights to generate representative samples.” (p.10, Methods section, revised manuscript)

*2) The authors rely on the DHS sampling frame to select the unit of aggregation for the sub-national index. In some countries, there are as few as 3 regions (e.g., Malawi) and in others, as many as 25 regions (e.g., Peru). Why did the authors not use second level administrative units for the sub-national indices, when available? It seems as though they may have used second level units for some of the heat maps (see below). Why the inconsistency between the admin unit used for the index and the heat maps for a given country (I understand why it might vary by country)?*

**Authors’ response:**

We used first-level administrative units to calculate the sub-national index for three reasons. First, data on first-level administrative units was available for all countries, whereas this was not the case for second-level administrative units. For our analysis, this implied that we had on average 12 first-level administrative units across all 55 countries, ranging from 3 regions in Malawi to 36 states and union territories in India. Second, the first-level administrative units included in the DHS are typically representative at the regional level or groups of regions, whereas this is not necessarily the case for second-level administrative units in the DHS. Third, indicators such as under-five mortality can be estimated precisely using a large number of observations for first-level administrative units.

“When calculating the child-based capability index at the regional level for all countries, we used first-level administrative units (as opposed to second-level administrative units) because: i) data on first-level administrative units was available for all countries in the DHS whereas this was not the case for lower-level administrative units; ii) for most DHS surveys, averages at these sub-national levels provided regionally representative estimates; and iii) the components of our index can be estimated precisely using a large number of observations for first-level administrative units.” (p.11, revised manuscript, Methods Section)

To examine time trends in child survival along the development spectrum, we also mapped results for selected countries which had both first- or second-level administrative boundaries in the DHS that have remained largely consistent over the study period. Malawi, for instance, had data on first- and second-level administrative boundaries that remained consistent over the study period (2000s to 2010s). To increase the resolution of the heat maps, we therefore used the lowest available administrative unit that was consistently available across DHS surveys rather than aggregating at first-level administrative units. In the case of Malawi, this implied that we were able to map child health along the development spectrum for all its second-level units (districts) within larger first-level administrative units.

“To examine time trends in under-five survival by maternal educational attainment and wealth, we show results for selected countries with first- or second-level administrative boundaries that have remained largely consistent over the study period (2000s to 2010s). To further increase the resolution of the heat maps, we used the lowest available administrative unit that is consistently available across the DHS country-surveys (either first- or second-level administrative units). As an example, we show child health along the development spectrum for the 36 second-level administrative units (states) and the Federal Capital Territory (FCT) of Nigeria – rather than aggregating by first-level administrative units – in 2003 and in 2013. This approach allowed us to examine sub-national shifts in child health for selected countries across the development spectrum and over time during the final run up towards the Millennium Development Goals.” (p.11, Methods section, revised manuscript).

*3) The authors point out repeatedly that the advantages of their sub-national index over that of Harttgen and Klasen [13] are that they don't use imputation and that child mortality is more sensitive than life expectancy. While I agree that using actual data over imputation is an advantage, the argument (and the paper) would be stronger if the authors made a direct comparison of the 2 methods and demonstrated their sensitivity advantage.*

**Authors’ response:**

Harttgen and Klasen used data from DHS surveys which were conducted earlier (2000s) and a direct comparison of our results is thus, unfortunately, not possible. As suggested by the reviewer, however, we can replicate the methods using the same data to demonstrate differences in sub-national results. To do so, we have replicated the household-based index developed by Harttgen and Klasen while taking the case of Zimbabwe DHS survey (2015) as an example. Our direct comparison of the two methods, displayed in Table S3, reveals two findings. First, we find differences in the rank of regions (the region Manicaland, for instance, performs worse on our child-based index, compared to the index developed by Harttgen and Klasen [difference in rank position: -2]. Second, our child-based index has a somewhat wider range (0.420 – 0.723), suggesting that our index may indeed pick up larger within-country differences.

“Table S3. Comparison of sub-national index with Harttgen and Klasen (2012).”

*Notes:* Table compares the child-based human development index (HDI) with the household-based human development index developed by Harttgen and Klasen (2012). Source: authors’ calculations using data from the Demographic and Health Survey for Zimbabwe (2015).

*Regarding the heat maps, more detail is needed to understand how they were created. What software package was used? Was there some kind of smoothing routine? If lower than first level administrative units were used, this should be specified. For example, Malawi has only had 3 regions - or 3 data points for all 3 dimensions. Presumably more than 3 units were used for the heat maps. Is this true?*

**Authors’ response:**

We thank the reviewer for bringing this to our attention. We have now further clarified the way the heat maps were created throughout the paper. First, we have mentioned the software program used to generate the figures in the Methods section. Specifically, we used the command *twoway contour* in Stata v15.1 (College Station, TX), which displays *z* as filled contours in (*y*,*x*). As mentioned by the reviewer, when (*z*, *y*, *x*) does not fill a regular grid, Stata uses the default thin-plate-spline (TPS) interpolation method. Briefly, TPS are a spline-based technique for data interpolation and smoothing (Duchon, 1976).

“Figures were generated using the *twoway contour* command in Stata MP 15.1 (College Station, TX), which displays z as filled contours in (y,x), using the default thin-plate-spline interpolation method [23].” (p.10, revised manuscript, Methods Section)

Second, we have now further specified in the Methods section that we used the lowest available administrative unit that is consistently available across the DHS country surveys for the heat maps (either first- or second-level administrative units). Malawi, for instance, had data on first- and second-level administrative boundaries that remained consistent over the study period (2000s to 2010s). To increase the resolution of the heat maps, we therefore used the lowest available administrative unit that was consistently available in the DHS surveys. In the case of Malawi, this implied that we were able to map results for all its second-level units (districts) within larger first-level administrative units.

“To examine time trends in under-five survival by maternal educational attainment and wealth, we show results for selected countries with first- or second-level administrative boundaries that have remained largely consistent over the study period (2000s to 2010s). To further increase the resolution of the heat maps, we used the lowest available administrative unit that is consistently available across the DHS country-surveys (either first- or second-level administrative units). As an example, we show child health along the development spectrum for the 36 second-level administrative units (states) and the Federal Capital Territory (FCT) of Nigeria – rather than aggregating by first-level administrative units – in 2003 and in 2013. This approach allowed us to examine sub-national shifts in child health for selected countries across the development spectrum and over time during the final run up towards the Millennium Development Goals.” (p.11, Methods section, revised manuscript).

Reference:

Duchon J. (1976), Splines minimizing rotation invariant semi-norms in Sobolev spaces. pp 85–100, In: Constructive Theory of Functions of Several Variables, Oberwolfach 1976, W. Schempp and K. Zeller, eds., Lecture Notes in Math., Vol. 571, Springer, Berlin.

*I also think that the heat maps would be more useful if they were overlaid with geographic regions within countries (i.e., with geospatial hot spot analysis). Take the example of the Philippines, which the authors point out has a persistent hot spot of high child mortality on the wealth and education dimensions. However, we don't know if the hot spot is in fact in the same geographic region by looking at the two heat maps. If we assume that it is, the next questions would be - why that region? Is it particularly isolated and difficult to get services to? Is it in an area of political instability and on-going conflict? It would be helpful if the authors went into more detail about how the heat maps would be used by a policy maker or program planner, and they could use the Philippines as an example.*

**Authors’ response:**

We thank the reviewer for this excellent suggestion. Since an analysis for all 55 countries in our study would be a relatively large undertaking, we therefore plotted results for geographical regions in a selected country, which is the recommended strategy by the reviewer. To map within-country variation, we used individual-level child-data from the DHS of the Philippines of 2017 (*N*=22,158 children). We constructed the child-based capability index for each region of the Philippines, and then grouped regions in ten groups of similar size (deciles) based on their index values. In Figure 2 in the paper, we now map the sub-national child-based capability index in the Philippines, which ranged from 0.295 in the Autonomous Region in Muslim Mindanao (dark red) to 0.693 in the National Capital (dark blue).

“Fourth, showing results for geographical regions may improve the usability and interpretation of our approach, and point decision-makers to more targeted efforts to increase child-based capabilities in at-risk regions. We therefore plotted the child-based capability index, as calculated in Equation 2, for geographical regions. Since such a geographical analysis for all 55 countries would be a relatively large undertaking, we show results for a selected country. To illustrate within-country variation, we used individual-level child-data from the DHS of the Philippines of 2017 (*N*=22,158 children). After constructing the child-based capability index for each geographical region of the Philippines, we grouped regions in ten groups of similar size (deciles) based on their index values. We then mapped the index for each of the deciles using maps provided by the Philippine Statistics Authority and National Mapping and Resource Information Authority.” (p.12, Methods section, revised manuscript).

“Figure 2. Mapping regional variation in child-based capabilities.”

*Notes:* Figure 2 shows the child-based capability index across regions in the Philippines. Source: authors’ calculations using child-data from the Philippines Demographic and Health Survey (2017) and data on geographic boundaries from the Philippine Statistics Authority and National Mapping and Resource Information Authority (*N*=22,158).

We also aim to build on the methods developed in this paper in future work, by conducting a geospatial analysis for several low- and middle-income countries using geo-referenced household surveys with data on child health, maternal education, and household wealth (LBD Under-5 Mortality Collaborators 2019). Our overarching goal is to provide detailed maps of where the different components of the index co-exist across low-resource settings to further guide “precision public health”. Showing results for geographical regions may improve the usability of our results, and point policy-makers and program implementers to more targeted efforts to increase child-based capabilities in at-risk regions.

Reference:

Local Burden of Disease Under-5 Mortality Collaborators (2019). Mapping 123 million neonatal, infant and child deaths between 2000 and 2017. *Nature*; 574(7778):353-358.

*Other comments 1) I'm confused as to why the authors write that this paper provides a better understanding of how wealth, education, and under-5 survival are "co-produced." Maybe they mean where these factors are co-produced or co-exist? It would be helpful to me (and perhaps other readers) if the authors could provide some clarification of this concept and/or more of a theoretical background for their intent in applying this term here.*

**Authors’ response:**

We thank the reviewer for these comments. The term “co-production” was initially coined to designate a process in which contributions from individuals who are not in the same organization are transformed into goods and services (Brudney & England 1983). In our paper, we used this term to describe the co-production of child health across government sectors (e.g., the education and health sectors) and households in low-resource settings. It is currently not evident, however, what counts as co-production in global health: what is being produced, under what circumstances, and by which organizations or individuals. We have therefore now removed this term and rephrased the corresponding sentences.

Reference:

Brudney JL, England RE (1983) Towards a definition of the co-production concept. *Public Administration Review* 43(1):59–65.

*2) The authors could be more accurate in describing the locations on the heat maps and avoid using household or individual level terms that imply something that they cannot really say about individual households or mothers. Since the maps reflect aggregates of within-country regions, they cannot, for example, tell us about poor households or uneducated mothers within an overall wealthy region. The text might read something like: low survival shifted from regions with more wealthy households to regions with more poor households.*

**Authors’ response:**

We thank the reviewer for this helpful comment. We have now carefully gone through the paper to further clarify that our results represent aggregates at the national and administrative unit levels.

“In Egypt and Malawi, for instance, low under-five survival shifted from areas with on average wealthier households to areas with on average poorer households.” (p.23, Discussion section, revised manuscript)

*3) I think that the paper could be tightened by cutting at least one of the long lists of countries, the additional list of administrative units, and avoiding repetition about the details of [13] that uses imputation. This would leave room in the introduction to explaining how the paper supports "co-production" of the 3 human capital components and in the discussion for how the heat maps can be used by policy-makers.*

**Authors’ response:**

First, we have now trimmed down the Introduction section, removing mentions of our findings and elements of discussion to the Discussion section to more concisely describe the study aims. We have also removed the long list of countries and administrative units from the Methods section to reduce redundancy (noting that we now refer to Tables 1 and 2, which contain similar information on the selected countries included in the analysis). We have also removed redundant details on comparisons with the methodological approaches used by other studies from the Methods and Discussion sections.

Second, we have now provided additional discussion on how our approach can be useful to other researchers and public health practitioners seeking to improve child-based capabilities in low-resource settings. To do so, we have provided more details on the construction of our estimates and heat maps in the Methods section. To further facilitate the presentation and dissemination of our results to policy-makers, we have illustrated how the child-based capability index can be mapped for geographical regions.

*4) In the methods, page 5, the description of the survival indicator needs re-organization. I would recommend moving the information on page 6 about birth history earlier and rephrasing the sentence: "…create a binary variable indicating whether the child was alive or not…" Which child? The wording is confusing without understanding the source of the child information. Also, please add the information about which women are eligible to be interviewed about their birth history in the DHS (i.e., their age).*

**Authors’ response:**

We have now moved information about the child-data in the Methods section before the descriptions of the outcome variables, and clarified the text to further facilitate the understanding of our outcomes.

“***Data Sources and Sample Population***

To illustrate the computation of our child-based capability index, we extracted data on age, sex, geographical location of household, under-five survival, maternal educational attainment, and household wealth from two DHS surveys (one carried out during the 2010s and, when available, one carried out during the 2000s) for 55 LMICs. A key advantage of the DHS is the availability of comparable data for multiple countries and consistent quality of reporting and data over time [16]. The country and survey selections were chosen to be illustrative rather than exhaustive: our aim was to include a mix of low- and lower-middle-income countries for which nationally and regionally representative data on our outcomes was available (our approach could easily be reproduced to a larger number of countries and years). The countries included in our analysis are listed in Table 1. For each survey, the DHS birth recode files provide a full birth history of all women interviewed during the survey and includes data for the mother of each child [16]. In DHS surveys, the birth history data are typically collected from all women ages 15–49 years old. We included all children born in the past ten years in a household surveyed by the DHS, and for whom complete data on survival status, maternal education, and household assets (wealth) was available.

***Underlying Outcome Variables: Health, Education, and Wealth***

We built on the HDI approach, hence we assembled three similar components toward construction of a child-based capability index. First, we selected maternal educational attainment, calculated as the highest grade or level of formal schooling attained (years of schooling) by the mother of the child (“Edu”). We used maternal education as opposed to average household or paternal education, since maternal education is a strong marker of child health, reflects large gender disparities across households in accessing formal education in LMICs, and is commonly available across surveys [17-19]. Second, we selected a child’s household wealth index, ranging from one to five (five being the wealthiest) (“Wealth”). Wealth quintile is a measure of household wealth relative to other households across countries (for national comparisons) or within-countries (for sub-national comparisons) [20, 21] and is based on ownership of household assets and quality of the dwelling [22]. The same household wealth quintile was assigned to all children living in the household surveyed by the DHS, and maternal educational attainment was assigned to all children from her birth history. Third, we created a binary variable indicating whether the child was alive or not at age 5 years at the time of the survey (“U5S”): under-five survival would mean a higher score on all three components would be considered positive (Figure S1). We avoided the use of imputation in generating all three outcome variables. Additional details on outcomes and sensitivity analyses are provided in Text S1-S2 in the supplementary webappendix.” (p.6, Methods section, revised manuscript)

*5) In the analysis section, top of page 9 - second sentence, did the authors mean to write "administrative unit" in the parentheses - not "household"?*

**Authors’ response:**

We thank the reviewer for bringing this to our attention and have now corrected the sentence:

“To increase the number of observations in each (administrative unit) cell, we calculated under-five survival, maternal educational attainment, and household wealth by the first-level administrative unit available in each DHS.” (p.10, Methods section, revised manuscript)

*6) In the analysis section, how were household wealth and maternal education assigned to individual children before aggregating? Presumably, the same household wealth indicator was assigned to all children in the household, and the maternal education to all children from her birth history record?*

**Authors’ response:**

As correctly noted by the reviewer, the same household wealth indicator was assigned to all children in the household, and maternal education was assigned to all children from her birth history. We have now further clarified these points in the Methods section of the paper, where we write:

“The same household wealth quintile was assigned to all children living in the household surveyed by the DHS, and maternal educational attainment was assigned to all children from her birth history.” (p.7, Methods section, revised manuscript)

*7) The authors went to a great deal of effort to run a number of important sensitivity analyses. I was interested in knowing more about what they learned from these. What can be learned from the fact that the results were relatively insensitive to a wide range of specifications?*

**Authors’ response:**

We assessed whether our findings were robust to alternative methodological specifications, including different specifications of our analytical sample, indicators, and method to calculate the combined index. To do so, we used alternative analytical samples and definitions for the index components. We restricted the sample of children to mothers aged 15–30 years since recent changes in the education sector would be reflected in younger rather than older women cohorts. We also constructed a subindex for each component based on alternative ‘goalpost’ (reference) values. We used maternal educational attainment of 15 years, for instance, as reference value. In our main analysis, we also constructed a measure of wealth that allowed comparisons of the child-based capability index across countries. As a sensitivity analysis, however, we presented results when instead using the DHS-provided wealth index.

Our main results were generally consistent across these alternative specifications, generating additional confidence in the robustness of our main findings. We found little evidence that our results are an arbitrary consequence of data quality, methodological choices or assumptions, rather than naturally present in our dataset. Issues such as limited sample size, age of mother, recall bias, and the method used to calculate under-five mortality in our data are unlikely to substantially change the overall picture. In addition to testing the study’s internal validity, these analyses generate confidence in the relevance and wide applicability of our methodological approach to other data sources and outcomes. We have now provided additional discussion on these analyses in the Results section of the paper.

“Our results were generally consistent across sensitivity analyses, including when using alternative outcomes, goalpost values, and sample specifications (Figures S2-S3 and Table S1). Potential concerns such as limited sample size, recall bias among mothers, and our methodological approach to calculate under-five mortality are unlikely to substantially affect our main findings.” (p.17, Results section, revised manuscript)

*8) Figure 1 - Notes - The description of the x and y components is reversed - wealth and education, not education and wealth.*

**Authors’ response:**

We thank the reviewer for bringing this to our attention and have now corrected the footnotes:

“Notes: Figure 1 shows three axes, including health (under-five survival), wealth (household wealth), and education (maternal education) using two DHS surveys (2000s and 2010s), separately for each country.” (p.16, Results section, revised manuscript)

*9) In the discussion, the authors present possible explanations for the surprising results from Egypt and Peru. Did they consider stratifying the child survival data by the sex of the child to examine if this finding applied equally to boy and girl children?*

**Authors’ response:**

We thank the reviewer for this helpful suggestion. We have now stratified the child-survival data by sex of the child. While girls tend to have a biological advantage in under-five mortality (Sawyer 2012), previous research suggests that girls may be disadvantaged by other factors in low-resource settings (such as lower access to resources and gender inequality). Indeed, when disaggregating our results by sex, we find that under-five mortality is more pronounced for girls compared to boys (Figure S4 now displays results for Egypt and Peru, by sex). Disaggregating our results along socio-demographic dimensions can help point policy-makers and program implementers to more targeted efforts to improve core human capabilities.

“Figure S4. Heterogeneity in Child-based Capabilities, by Sex”

*Notes:* Figure shows three axes, including health (under-five survival), wealth (household wealth), and education (maternal education), using the most recently available DHS survey for Egypt and Peru, by a child’s sex.

Reference:

Sawyer CC (2012). Child mortality estimation: estimating sex differences in childhood mortality since the 1970s. *PLoS Medicine*; 9(8):e1001287.

*10) I think the authors might note that their sub-national index still masks within administrative unit variation. States in India are as big as some countries with significant within state variation.*

**Authors’ response:**

We agree with the reviewer and have now further clarified this point in the Results section, as well as in the limitations paragraph in the Discussion section of the paper, where we write:

“Second, we aggregated child outcomes using first- or second-level administrative units that were available in the DHS surveys. In the future, a more granular look at the HDI may improve the resolution of our findings (e.g., at the village level). Our results for the sub-national child-based capability index in India, for instance, may mask substantial heterogeneity within states and union territories.” (p.23, Discussion section, revised manuscript)

*Reviewer #2:*

*This is a useful and well-conducted study on the development of a child-based Human Development Index (HDI). The study design, datasets, and statistical methods and analyses are mostly adequate and of a good standard. The development of the HDI formula is relatively simple and straightforward although it's a bit debatable to use only maternal education as the education indicator. The authors claimed that the proposed HDI provides more detailed heterogeneity in child health within and across countries over time, which seems fine.*

**Authors’ response:**

We thank the reviewer for these comments.

*However, there are still a few issues needing attention. 1) The heatmap is a bit difficult to read and follow. The usability, visualisation and interpretation is very important for an index to be accepted and widely used. An online tool / interactive website for the proposed HDI with all instructions and explanations would be useful to showcase and promote the index.*

**Authors’ response:**

While creating – and maintaining – a comprehensive online tool and interactive website would require substantial additional resources, we have improved our manuscript in a number of ways to further facilitate its acceptance and dissemination to other researchers and decision-makers. First, we have clarified our data sources and methodological approach. We have provided more details, for instance, on the construction of our estimates and heat maps in the Methods section. Second, we have further improved the presentation of our key findings. We have illustrated, for instance, how the child-based capability index can be mapped for geographical regions (now shown in Figure 2).

Third, we aim to build on the methods developed in this paper in future work, by conducting a geospatial analysis for several low- and middle-income countries using geo-referenced household surveys with data on child health, maternal education, and household wealth (LBD Under-5 Mortality Collaborators 2019). Our overarching goal is to provide detailed maps of where the different components of the index co-exist across low-resource settings to further guide “precision public health”. Showing results for geographical regions may improve the usability of our results, and point policy-makers and program implementers to more targeted efforts to increase child-based capabilities in at-risk regions.

“Figure 2. Mapping regional variation in child-based capabilities.”

Reference:

Local Burden of Disease Under-5 Mortality Collaborators (2019). Mapping 123 million neonatal, infant and child deaths between 2000 and 2017. *Nature*; 574(7778):353-358.

*2) Some form of validation of the index would be helpful. How do we know the proposed index placed among other similar/available HDIs? similarities and differences? A side by side comparison with other indices would be very helpful. Also, although there are 55 countries, can authors please take one country out to comprehensively illustrate and explain the usefulness of the index?*

**Authors’ response:**

First, we now show side-by-side comparisons with other commonly used indices at both the national and sub-national levels. At the national level, we compare our child-based capability index with the UNDP’s Human Development Index (HDI), the World Bank’s Human Capital Index (HCI), and the Socio-Demographic Index (SDI) developed by the Global Burden of Disease study. We show index values and rankings for each to allow direct comparisons of differences in ranking, as well as differences in the absolute values and range of the index. We find that the correlation between our child-based index and these indices at the national level was highest for the SDI (0.96, *p*-value < 0.01), whereas it was lowest for the HCI (0.84, *p*-value < 0.01) (Table S2). At the sub-national level, we now also show direct comparisons with a household-based index developed by Harttgen and Klasen (2012) (Table S3).

“Text S3. Comparison with other Indices” (Appendix, revised manuscript)

“Table S2. Comparison with other National-level Indices” (Appendix, revised manuscript)

“Table S3. Comparison with other sub-National Index” (Appendix, revised manuscript)

Second, we now show results from a supplementary analysis to comprehensively illustrate the policy relevance of the child-based capability index. Since an analysis for all 55 countries in our study would be a relatively large undertaking, we plotted results for geographical regions in a selected country, which is the recommended strategy by the reviewer. To map within-country variation, we used individual-level child-data from the DHS of the Philippines of 2017 (*N*=22,158 children). We constructed the child-based capability index for each region of the Philippines, and then grouped regions in ten groups of similar size (deciles) based on their index values. In Figure 2 in the paper, we now map the sub-national child-based capability index in the Philippines, which ranged from 0.295 in the Autonomous Region in Muslim Mindanao (dark red) to 0.693 in the National Capital (dark blue).

“Fourth, showing results for geographical regions may improve the usability and interpretation of our approach, and point decision-makers to more targeted efforts to increase child-based capabilities in at-risk regions. We therefore plotted the child-based capability index, as calculated in Equation 2, for geographical regions. Since a geographical analysis for all 55 countries would be a relatively large undertaking, we show results for a selected country. To illustrate within-country variation, we used individual-level child-data from the DHS of the Philippines of 2017 (*N*=22,158 children). After constructing the child-based capability index for each geographical region of the Philippines, we grouped regions in ten groups of similar size (deciles) based on their index values. We then mapped the index for each of the deciles using maps provided by the Philippine Statistics Authority and National Mapping and Resource Information Authority.” (p.12, Methods section, revised manuscript).

“Figure 2. Mapping regional variation in child-based capabilities.”

*3) Overall, the discussion is a bit too brief. Need to be more comprehensive and critical.*

**Authors’ response:**

To address comments by the editors and reviewers, we have now restructured the Discussion section so that the first paragraph consists predominantly of a summary of the findings, and removed redundant details on comparisons with the methodological approach used by Harttgen and Klasen 2012. We have now also provided additional discussion on comparisons with other commonly used indices, and how our approach can be used and replicated by other researchers and public health practitioners seeking to bolster child-based capabilities in at-risk regions. We have also substantially expanded the paragraph on study limitations in the Discussion section to provide a more comprehensive and critical stance.

“Our approach relied on fewer assumptions and is methodologically straightforward to extend to other settings, outcomes, and populations [34]. We illustrated it with a limited number of countries, though it can easily be reproduced in other settings using, for instance, Multiple Indicator Cluster Surveys (MICS) data [35]. Likewise, it can be replicated with alternative outcomes (e.g., stunting, wasting) to examine progress along the development spectrum with specific prevention programs within and across countries (e.g., nutrition programs, vaccination coverage). Our results can also be mapped for geographical regions within countries (as illustrated in Figure 2), to point decision-makers and public health practitioners to more targeted efforts to improve outcomes among populations in at-risk regions [36].” (p.25, Discussion section, revised manuscript).

“Second, we aggregated child outcomes using first- or second-level administrative units that were available in the DHS surveys. In the future, a more granular look at the HDI may improve the resolution of our findings (e.g., at the village level). Our results for the sub-national child-based capability index in India, for instance, may mask substantial heterogeneity within states and union territories.” (p.25, Discussion section, revised manuscript).

“Third, nationally representative household surveys are a relatively expensive and infrequent source of detailed population data [16]. Future research efforts are needed to determine whether alternative approaches are feasible to estimate the different components of the child-based capability index more frequently and economically. Machine learning techniques, for instance, have been recently applied to data from mobile phones, social media, and satellites to estimate socio-economic indicators, including population changes [40] and household wealth [41].” (p.25, Discussion section, revised manuscript).” (p.25, Discussion section, revised manuscript).

*Reviewer #3:*

*The topic of this paper is interesting. The paper was written well.*

**Authors’ response:**

We thank the reviewer for these comments.

*However, I did not find important implications of the proposed method. First, it requires individual data that not every country can have for every year.*

**Authors’ response:**

We thank the reviewer for these comments. We feel that the methods proposed in our paper have a number of key implications. First, we show, for the first time, heat maps of health, education, and wealth, and identifies areas of high under-five mortality stratified by wealth and parental education for multiple low- and middle-income countries. Second, we use measures of human development that are sensitive indicators of population-level social and economic inequalities and are likely to be highly policy-relevant. In doing so, we avoid the use of imputation to generate under-five mortality. Third, we estimate an aggregate index that can be used to target and track changes in child capabilities across and within countries over time. Our methodological approach is straightforward to implement by other researchers and public health practitioners and can be easily adapted to other indicators, datasets, and populations.

Nevertheless, as pointed out by the reviewer, the proposed method comes with a number of trade-offs. Household surveys, for instance, are a relatively expensive and infrequent source of detailed population data. The DHS program are conducted approximately every 5 years and certain areas may be unreachable because of e.g., political instability or conflict. Further research is needed to determine whether alternative approaches are feasible to estimate the components of the index more frequently and economically. Machine learning techniques, for instance, have been recently applied to existing data from mobile phones, social media, and satellites to estimate socio-economic indicators such as population changes (Deville et al., 2014), wealth (Jean et al., 2016), and education (Sekara et al., 2018).

“Third, nationally representative household surveys are a relatively expensive and infrequent source of detailed population data [16]. Future research efforts are needed to determine whether alternative approaches are feasible to estimate the different components of the child-based capability index more frequently and economically. Machine learning techniques, for instance, have been recently applied to data from mobile phones, social media, and satellites to estimate socio-economic indicators, including population changes [40] and household wealth [41].” (p.25, Discussion section, revised manuscript).” (p.25, Discussion section, revised manuscript).

References:

Deville P, Linard C, Martin S, Gilbert M, Stevens FR, Gaughan AE, Blondel VD, Tatem AJ. Dynamic population mapping using mobile phone data. *PNAS*. 2014 Nov 11;111(45):15888-93.

Jean N, Burke M, Xie M, Davis WM, Lobell DB, Ermon S. Combining satellite imagery and machine learning to predict poverty. *Science*. 2016 Aug 19;353(6301):790-4

*Second, how to measure household wealth to be comparable between urban and rural, across regions of a country and among country is challenging. The current method used based on ownership of household assets and quality of the dwelling is controversy.*

**Authors’ response:**

As pointed out by the reviewer, while direct estimates of wealth are widely available in most high-income settings, this is often not true in low- and middle-income countries, where a majority of the rural population engages in subsistence farming, and many urban households depend on informal employment with irregular incomes. Given these limitations, most national household surveys rely on household assets to generate household wealth quintiles, which allow comparisons of health measures across equally sized groups within a given population. For comparisons of wealth and the combined index across countries, we therefore constructed an international household asset index (Smits & Steendijk 2014).

We also conducted sensitivity analyses to generate additional confidence in the robustness of our findings. We used the wealth index built into the DHS surveys, for instance, which takes into account country-specific differences in household assets and quality of the dwelling. Our measure of household wealth can be applied in a straightforward manner by others seeking to replicate our work. We also considered using predicted absolute incomes by simulating household income from an asset index and macroeconomic data. This approach, however, would have relied on additional assumptions, including that the income distributions are log-normally distributed, and that individual ranks of households are the same in the income distribution as they are in the distribution of the asset index (Fink et al, 2017).

“In our main analysis, we constructed an international household asset index that allowed comparisons of the child-based capability index across countries [21]. This approach however makes the index somewhat more complex to implement by public health practitioners. We therefore present national-level results using the wealth index built into the DHS. The DHS wealth index takes into account country-specific differences and can be extended in a straightforward manner to other countries and periods. We also considered using predicted absolute incomes to measure household wealth by simulating household income from an asset index and macroeconomic data [20], but which would have relied on additional assumptions.” (Text S2, revised manuscript)

References:

Smits J, Steendijk R (2014). The International Wealth Index. *Social Indicators Research*. 122(1):65-85.

Fink G, Victora CG, Harttgen K, Vollmer S, Vidaletti LP, Barros AJD (2017). Measuring Socioeconomic Inequalities With Predicted Absolute Incomes Rather Than Wealth Quintiles: A Comparative Assessment Using Child Stunting Data From National Surveys. *Am J Public Health*. 107(4):550-5.

Poirier MJP, Grignon M, Grépin KA, Dion ML (2018). Transnational wealth-related health inequality measurement. *SSM - Population Health*;6:259-75.

*Third, that health is based on only one indicator might not be sensitive enough to detect small differences among countries.*

**Authors’ response:**

We thank the reviewer for this comment. We aim to keep the combined index ‘as simple as possible’ for three reasons. First, we seek to reduce redundancies related to a multidimensional index. The different components of the child-based capability index are strongly correlated with each other empirically. Adding indicators may question the “efficiency” of the index, so that the combined index might not reveal much more than its single components (Ghislandi et al, 2019). Second, we use data that can be sufficiently stable as to allow comparisons across countries and time. Adding indicators may require additional resources and assumptions (such as around measurement error). Third, increased complexity in the construction of the index may reduce the usability of the index, which is important for an index to be adopted widely to decision-makers and program implementers working in low-resource settings.

Reference:

Ghislandi S, Sanderson W, Scherbov S (2019). A Simple Measure of Human Development: the Human Life Indicator. *Population and Development Review* 45 (1): 219-233.
