# Supplementary material for: Nationally and regionally representative analysis of 1.65 million children aged under 5 years using a child-based human development index: A multi-country cross-sectional study
Source: PLoS Med. 2020 Mar 16;17(3):e1003054. doi: 10.1371/journal.pmed.1003054 (PMC7075547; doi:10.1371/journal.pmed.1003054)
Supplement: Attachment — Submitted filename: HDI Response 30 Jan 2020.docx [file pmed.1003054.s014.docx]

**Editors’ comments**

*Please round the total number of 1,657,272 children down to 1.65 million in your title and Author Summary. Please use the accurate total number of 1,657,272 children throughout the main text.*

**Authors’ response:**

We have now rounded the total number of 1,657,272 children down to 1.65 million in the title and Author Summary and have used the total number of 1,657,272 children in the main text as recommended.

“Nationally and regionally representative analysis of 1.65 million children aged under 5 years using a child-based human development index: a multi-country cross-sectional study” (Title, revised manuscript)

*Please remove spaces between refs in square brackets where more than one are cited.*

**Authors’ response:**

We have now removed spaces between references in square brackets where more than one are cited.

*Please revise your title to "Nationally and regionally representative analysis of 1.65 million children aged under 5 years using a child-based human development index: a multi-country cross-sectional study". Apologies for another minor revision to your title.*

**Authors’ response:**

We thank the editors for this excellent suggestion and have now revised the title accordingly.

“Nationally and regionally representative analysis of 1.65 million children aged under 5 years using a child-based human development index: a multi-country cross-sectional study” (Title, revised manuscript)

*Abstract background - please define what is meant by ‘human capabilities’*

**Authors’ response:**

We have now further clarified the term human capabilities in the background section of the abstract.

“Background: Education and health are both constituents of human capital that enable people to earn higher wages and enhance people’s capabilities. Human capabilities may provide an opportunity to lead fulfilling lives by achieving a valuable combination of human functionings – i.e., what people are able to do or be as a result of their capabilities. A better understanding of how these different human capabilities are produced together could point to opportunities to help jointly reduce the wide disparities in health and education across populations.” (Abstract, revised manuscript)

*Abstract methods and findings: Please make clear the range of scores that can be generated by the child-based HDI; whether these are normalized.*

**Authors’ response:**

We have now provided additional information on the construction of the index in the methods and findings sections of the abstract, and included the range of potential scores generated by the index.

“We normalize the range of each component using data on the minimum and maximum values across countries (for national comparisons) or first-level administrative units (for sub-national comparisons). The scores that can be generated by the child-based HDI range between 0 and 1.” (Abstract, revised manuscript)

*Abstract conclusions - Line 45: Please add ‘to our knowledge’ or similar to avoid assertions of primacy*

**Authors’ response:**

“This study maps patterns and trends in human capabilities and is among the first, to our knowledge, to introduce a child-based HDI at the national and sub-national level.” (Abstract, revised manuscript)

*Author Summary: Please remove border from Author Summary.*

**Authors’ response:**

We have now removed the border from the Author Summary.

*Please revise the first two bullet points of ‘Why was this study done?’ to use non-identical language to your abstract/introduction.*

**Authors’ response:**

We have now revised the first two bullet points to use non-identical language.

“Education and health are aspects of human capital which may have important economic benefits for individuals and households. These human capabilities reflect a person’s freedom to choose different ways of living and lead a fulfilling life.

Highlighting how different human capabilities are produced together can support efforts to synergistically reduce the wide disparities in health and education across populations.” (Author Summary, revised manuscript)

*In the first bullet point of ‘What did the researchers do and find?’, please revise to ‘1.65 million children under five years of age’*

**Authors’ response:**

We have revised the first bullet point as recommended by the editors.

“We use nationally and regionally representative individual-level data from 1.65 million under-five children in 55 low- and middle-income countries to examine patterns in human capabilities at the national and regional levels, between 2000 and 2017. ” (Author Summary, revised manuscript)

*In the first bullet point of ‘What do these findings mean?’, please clarify what is meant by ‘child capabilities’*

**Authors’ response:**

We have now replaced the term “child capabilities” with the term “human capabilities” which is more consistent with the abstract and the previous bullet points listed in the Author Summary.

“Areas of chronic deprivation across and within countries may require alternative policy approaches to improving human capabilities jointly in low resource settings.” (Author Summary, revised manuscript)

*In the second bullet point of the same, please revise to ‘These findings may point decision-makers’*

**Authors’ response:**

We have revised the sentence accordingly.

“These findings may point decision-makers to more targeted efforts to further reduce persistent health disparities toward the Sustainable Development Goals.” (Author Summary, revised manuscript)

*Please revise ‘persistent health disparities toward the Sustainable Development Goals.’*

**Authors’ response:**

We have now revised the sentence accordingly.

“These findings may point decision-makers working towards achieving the Sustainable Development Goals to more targeted efforts to further reduce persistent health disparities.” (Author Summary, revised manuscript)

*In the final bullet point of ‘What do these findings mean?’, please describe the main limitations of your study.*

**Authors’ response:**

We have now added the main limitations of our approach in the final bullet point as recommended.

“Limitations of our study include its descriptive nature, lack of information beyond first- and second-level administrative units, and limited generalizability beyond the countries analyzed.” (Author Summary, revised manuscript)

*Introduction. Please indicate in your Introduction whether your study is novel and how you determined that.*

**Authors’ response:**

We have now further clarified the key novelty of our study and how we determined this.

“A few studies have aimed to calculate an HDI for sub-national units within country income groups using a regression-based approach and an asset index to impute income [10,11], by internal migration status [12], as well as using household-level data for multiple low- and middle-income countries (LMICs) [13,14]. One challenge with calculating health or mortality outcomes at the sub-national or household level is that there may be limitations due to small sample size and limited variation without a continuous variable (i.e., since in most households either none, one, or two members died, resulting in household mortality rates clustered around zero or values such as 0.50). To address this issue, a handful of studies have calculated mortality rates using imputation methods. One study used Demographic and Health Surveys (DHS) data to impute child mortality by regressing child mortality on household and community socioeconomic characteristics, applying life table systems [15] to estimate household-specific life expectancy at birth, and subsequently calculating a health index using estimated life expectancy for each household [13].

In this study, we aim to make two contributions to the literature on human development and capabilities, while taking the illustrative example of under-five mortality. First, we used nationally and regionally representative individual-level data from 55 LMICs to show current patterns as well as trends in human capabilities at the national and regional levels. Second, we used under-five mortality, maternal educational attainment and household wealth as our measures of health, education, and wealth, respectively, to introduce a novel child-based capability index (i.e., a child-based version of the HDI). The premise of the study was not to replace existing measures of human development, but rather to explore measures that are particularly sensitive to population-level social and economic inequalities, and are likely to be highly policy-relevant [16-18]. In doing so, this study aimed to compute a child-based capability index which is straightforward to implement as a summary metric for decision-makers seeking to bolster human capabilities in the post-2015 development era, and could be easily adapted to other indicators, populations, and settings.” (p.5, Introduction section, revised manuscript)

*Line 64 - please revise to ‘was initially calculated’*

**Authors’ response:**

We have now revised the sentence to ‘was initially calculated’.

“In 1990, a Human Development Index (HDI) was developed as an alternative to the traditional unidimensional economic measure of development (e.g. the gross domestic product or GDP [8]) and was initially calculated as the arithmetic mean of normalized values of life expectancy, educational attainment, and income [9].” (p.4, Introduction section, revised manuscript)

*Please remove the study results and/or conclusion from the Introduction, and conclude the Introduction with a clear description of the study question or hypothesis.*

**Authors’ response:**

We have now removed the study results and conclusion from the Introduction section of the paper and have further clarified the key study aims as recommended.

“In this study, we aim to make two contributions to the literature on human development and capabilities, while taking the illustrative example of under-five mortality. First, we used nationally and regionally representative individual-level data from 55 LMICs to show current patterns as well as trends in human capabilities at the national and regional levels. Second, we used under-five mortality, maternal educational attainment and household wealth as our measures of health, education, and wealth, respectively, to introduce a novel child-based capability index (i.e., a child-based version of the HDI). The premise of the study was not to replace existing measures of human development, but rather to explore measures that are particularly sensitive to population-level social and economic inequalities, and are likely to be highly policy-relevant [16-18]. In doing so, this study aimed to compute a child-based capability index which is straightforward to implement as a summary metric for decision-makers seeking to bolster human capabilities in the post-2015 development era, and could be easily adapted to other indicators, populations, and settings.” (p.5, Introduction section, revised manuscript)

*Line 90 - please remove sentence beginning ‘We show heat maps of health…’;*

**Authors’ response:**

We have now removed the sentence as recommended by the editors.

*Line 94 - please remove sentence beginning ‘Our approach to compute…’ (more appropriate for Discussion)*

**Authors’ response:**

We have now removed the sentence as recommended by the editors.

*Methods. Thank you for providing a completed RECORD statement. Please add the following statement, or similar, early in the Methods section: "This study is reported as per the REporting of studies Conducted using Observational Routinely-collected Data (RECORD) guideline (S1 Checklist)."*

**Authors’ response:**

We have now added the recommended sentence at the beginning of the Methods section.

“This study is reported as per the REporting of studies Conducted using Observational Routinely-collected Data (RECORD) guideline (S1 Checklist)” (p.6, Methods section, revised manuscript)

*Please refer to your prospective protocol / analysis plan early in the Methods section. Please indicate whether any changes (including those made in response to peer review comments) were made to the plan, with rationale.*

**Authors’ response:**

We have now referred to the prospective protocol early in the Methods section of the paper.

“There was a written prospective protocol for the study (S1 File), which was adapted in response to peer review comments to further clarify our methodological approach and results.” (p.6, Methods section, revised manuscript)

*Please clarify how data on ‘all children born in the past ten years’ relates to children under five years of age, as is the focus of your study.*

**Authors’ response:**

We thank the editors for this comment and have now further clarified the sentence.

“We included all children born in a household surveyed by the DHS, and for whom complete data on under-five survival status, maternal education, and household assets (wealth) was available. In our main analysis, we included under-five survival data for all children born in the past ten years preceding the survey. We considered alternative sample specifications in sensitivity analyses described below.” (p.6, Methods section, revised manuscript)

*Results. Line 268 - please include that results in Figure 1 are only displayed for selected countries*

**Authors’ response:**

We have now revised the sentence accordingly:

“In Figure 1, we display normalized under-five survival, maternal education, and household wealth ranging from 0 (worst) to 1 (best) for selected countries.” (p.14, Results section, revised manuscript)

*Line 270 - please consider an alternative term for ‘depth’ (of under-five survival)*

**Authors’ response:**

We have now replaced the term ‘depth’ with ‘level’.

“The color in the figure indicates the level of under-five survival.” (p.14, Results section, revised manuscript)

*Line 278 - please revise to ‘on average’*

**Authors’ response:**

We have now revised the sentence accordingly.

“In Malawi in the 2000s, for instance, poor child health (red zones in the figure) was concentrated in areas with relatively high on average levels of household wealth.” (p.15, Results section, revised manuscript)

*Discussion. Line 350 - Sentence beginning ‘In Colombia, Egypt, and Nigeria…’ does not seem to naturally follow on from previous sentence, outlining that improvements over time do not occur in all countries. Please clarify.*

**Authors’ response:**

We thank the editors for this comment and have now clarified the sentence.

“Second, our study shows trends in child health over time. We find improvements over time overall between 2000 and 2017, although this is not the case for all countries included in our study. In Cambodia and Nigeria, for instance, under-five survival improved in geographical areas at most levels of average maternal educational attainment and household wealth; whereas in Peru and the Philippines, for instance, under-five survival was distributed relatively consistently over time.” (p.21, Discussion section, revised manuscript)

*Line 388 - please remove ‘also’*

**Authors’ response:**

We thank the editors for bringing this to our attention and have corrected the sentence.

“Despite the overwhelming evidence of the associations between the core dimensions of human capabilities, few comprehensive measures presently exist to track investments in all three dimensions of the HDI jointly.” (p.23, Discussion section, revised manuscript)

*Line 415 - please begin your one-paragraph conclusion with ‘In conclusion’. Please add ‘to our knowledge’ or similar to avoid assertions of primacy.*

**Authors’ response:**

We have now added ‘In conclusion’ and rephrased the sentence to avoid assertions of primacy.

“In conclusion, this study maps patterns and trends in human capabilities and is among the first, to our knowledge, to introduce a child-based capability index building on the HDI.” (p.24, Discussion section, revised manuscript)

*Line 218 - please revise to ‘These findings may point decision-makers...’*

**Authors’ response:**

We have now revised the sentence as recommended.

“Fourth, showing results for geographical regions may improve the usability and interpretation of our approach, and may point decision-makers to more targeted efforts to increase child-based capabilities in at-risk regions.” (p.12, Methods section, revised manuscript)

*Text S3 - where p values are given, please specify the statistical test used*

**Authors’ response:**

We have now added the test used as recommended (noting that Text S3 has now become Text S2)

“We find that the correlation between our child-based index and these commonly used indices at the national level was highest for the SDI (*r* = 0.96, *p*-value for test of *r* = 0 < 0.01) and lowest for the HCI (*r* = 0.84, *p*-value for test of *r* = 0 < 0.01) (Table S2).” (Text S2, revised manuscript)

**Reviewers’ comments**

*Reviewer #1: The authors responded very well to previous comments and this paper has much improved as a result of their additional efforts. I only have two minor remaining comments:*

**Authors’ response:**

We thank the reviewer for these comments.

*The last paragraph in the introduction needs some re-working to clarify the aims and how the gap discussed in the first 2 paragraphs are meant to be filled. For example, it is never explicitly stated that the authors aimed to create an index in this paragraph to address issues with the existing indices. The sentence: "Second, we used under-five mortality, household wealth, and maternal educational attainment at the sub-national level as our measures of health, wealth, and education, respectively" leaves us hanging - they used these measures to do what?*

**Authors’ response:**

We thank the reviewer for this comment and have now further clarified the last paragraph in the Introduction section of the revised manuscript. Specifically, we have further highlighted the study’s aims and explicitly stated that we aimed to create a new index as recommended by the reviewer.

“In this study, we aim to make two contributions to the literature on human development and capabilities, while taking the illustrative example of under-five mortality. First, we used nationally and regionally representative individual-level data from 55 LMICs to show current patterns as well as trends in human capabilities at the national and regional levels. Second, we used under-five mortality, maternal educational attainment and household wealth as our measures of health, education, and wealth, respectively, to introduce a novel child-based capability index (i.e., a child-based version of the HDI). The premise of the study was not to replace existing measures of human development, but rather to explore measures that are particularly sensitive to population-level social and economic inequalities, and are likely to be highly policy-relevant [16-18]. In doing so, this study aimed to compute a child-based capability index which is straightforward to implement as a summary metric for decision-makers seeking to bolster human capabilities in the post-2015 development era, and could be easily adapted to other indicators, populations, and settings.” (p.5, Introduction section, revised manuscript)

*The word "data" is plural. Sentences with the clause "…survey data was available…" should be "…survey data were available…"*

**Authors’ response:**

We thank the reviewer for bringing this to our attention and have corrected the sentence.

“We developed the case study of a child-based capability index, and in this section, we present the main steps underlying its construction, which we then illustrate by the application to a selection of LMICs where household survey data were available.” (p.5, Methods section, revised manuscript)

*Reviewer #2: Thanks authors for their great effort to improve the manuscript.*

**Authors’ response:**

We thank the reviewer for these comments.

*I am mostly satisfied with the response and revision. However, for my 2nd point on validation and comparison with other existing indices, the response was satisfactory but didn't appear in the main text of the paper. The validation and comparison is a vital part of the paper so it should be mentioned and explained explicitly in the results section in the main paper. I can see this was explained in details in supplementary information Text S3 but it should appear or be mentioned in the main paper, as least briefly.*

**Authors’ response:**

We have now mentioned the analyses on validation and comparisons with other commonly used indices in the main text as recommended by the reviewer (noting that Text S3 has now become Text S2).

“Sixth, we compared our child-based capability index with other commonly used indices at both the national and sub-national level. At the national level, for instance, we compared our child-based capability index to the HDI, the World Bank’s Human Capital Index [29], and the Socio-Demographic Index developed by the Global Burden of Disease study [30].” (p.13, Methods section, revised manuscript)

“In S2 Table and S3 Table in the supplementary webappendix, we present results for side-by-side comparisons with other commonly used indices. We find that the correlation with our child-based capability index was highest for the Socio-Demographic Index and lowest for the Human Capital Index (Pearson's correlation coefficients = 0.96 and 0.84, respectively; *p*-values for tests of independence < 0.01).” (p.17, Results section, revised manuscript)

**Production issues**

*FIGURES and GRAPHICS: 1. Please ensure that all main figures are uploaded in .tif or .eps format. For more information about how to convert your figure files, please see our guidelines: http://journals.plos.org/plosmedicine/s/figures.*

**Authors’ response:**

We have uploaded all main figures in .tif format (i.e., Figures 1 and 2).

*2. Please remove embedded figures from your manuscript file and ensure the most up to date versions of your figures are uploaded to our submission system as separate TIF or EPS files. Please do not remove the figure legends and captions.*

**Authors’ response:**

We have removed embedded figures from the manuscript file and uploaded them separately as files.

*3. Please ensure that all main figure files are cited in ascending numerical order in the main text of the article.*

**Authors’ response:**

We have verified that all main figure files are cited in ascending numerical order in the main text.

*4. Please ensure that all main figures are referenced in the manuscript as Fig 1, Fig 2, etc. (including capitalization), rather than Figure 1, Figure 2, etc. Please note, however, that the file names themselves must not include the space, i.e. Fig1.tif, Fig2.tif, etc.*

**Authors’ response:**

We have carefully reviewed the entire paper and edited the references to main figures in the manuscript as Fig 1, Fig 2, etc. (including capitalization) rather than as Figure 1, Figure 2, and so on.

*5. Please ensure that all main figure legends are placed after the paragraph containing their first in-text citation.*

**Authors’ response:**

All main figure legends are now placed after the paragraph containing their first in-text citation.

*6. Please include the location from which you obtained the base map used in Fig 2 in the legends for this figure. Please note that, because all PLOS articles are published under a CC BY license (creativecommons.org/licenses/by/4.0/), we cannot publish proprietary maps such as Google Maps, Mapquest or other copyrighted maps. If your map was obtained from a copyrighted source please amend the figure so that the base map used is from an openly available source. The following websites are good examples of where you can source open access or public domain maps:*

** World of Maps - Many, but not all, of the maps are in the public domain so please check the usage rights before downloading. (http://www.worldofmaps.net)*

** U.S. Geological Survey (USGS) - All maps are in the public domain. (http://www.usgs.gov)*

** PlaniGlobe - All maps are published under a Creative Commons license so please cite “PlaniGlobe, http://www.planiglobe.com, CC BY 2.0” in the image credit after the caption. (http://www.planiglobe.com/?lang=enl)*

** Natural Earth - All maps are public domain. (http://www.naturalearthdata.com/about/terms-of-use/)*

**Authors’ response:**

We thank the production assistant for these helpful suggestions and have now used a base map from an openly available source – Natural Earth – which is one of the recommended sources.

“We then mapped the index for each of the deciles using a base map with geographic boundaries of the Philippines provided by Natural Earth (<https://www.naturalearthdata.com/>).” (p. 12, Methods section, revised manuscript)

“Source: authors’ calculations using child-data from the Philippines Demographic and Health Survey (2017) and a base map provided by Natural Earth (<https://www.naturalearthdata.com/>).” (Fig 2, revised manuscript)

*TABLES and BOXES:1. Please cite main tables in ascending numerical order in your main article file.*

**Authors’ response:**

We have verified that all tables are cited in ascending numerical order.

*2. Please ensure that all main tables in their entirety (including titles and footnotes), are placed after the paragraph containing their first in-text citation.*

**Authors’ response:**

All main tables in their entirety are placed after the paragraph containing their first in-text citation.

*METADATA: 1. Please ensure that the funders and grant numbers match between the Financial Disclosure field and the Funding Information tab in your submission form. Note that the funders must be provided in the same order in both places as well. Currently, your Financial Disclosure indicates that you received no specific funding for this work, though the Funding Tab appears to list funders.*

**Authors’ response:**

We have carefully reviewed all funding information and edited the funders and grant numbers to make sure that they match between the Financial Disclosure field and the Funding Information tab.

*2. In accordance with PLOS' data policy, please ensure that your Data Availability Statement in the submission form clearly identifies how readers can access your data. Data cannot be available on request, or only accessible by contacting one of the authors. Note that your Data Availability Statement will be typeset as it is written, so please ensure it is in complete sentences and appears as you would like it to in the published version. For details on our data policy, please see http://journals.plos.org/plosmedicine/s/data-availability*

**Authors’ response:**

We have verified that our data availability statement complies with the journal’s data policy.

“DHS survey data are available from the DHS Program (https://dhsprogram.com/) and all other study data are included in the paper and its supporting files.” (online submission system)

*ARTICLE FILE: 1. Please indicate the corresponding author in the byline by placing an asterisk (*) after their affiliation number.*

**Authors’ response:**

We have indicated the corresponding author (i.e., Jan-Walter De Neve).

*2. Please include the corresponding author's email address on the title page of your manuscript, indicated by an asterisk (*). Only an asterisk and the email address itself are required.*

**Authors’ response:**

We have indicated the corresponding author’s email address (i.e., janwalter.deneve@uni-heidelberg.de).

*3. Please remove author degree titles from the byline of your article file.*

**Authors’ response:**

We have now removed all author degree titles from the byline of the article file.

*4. Please ensure that all authors have at least one affiliation listed on the title page, and that all listed affiliations are attributed to at least one author. Affiliations should be listed in ascending numerical order in both the affiliation list and in the author byline.*

**Authors’ response:**

All authors have one affiliation listed and all listed affiliations are attributed to at least one author.

*5. Please ensure that your Ethics statement is available in the Methods section of your manuscript in its entirety.*

**Authors’ response:**

We have verified that the Ethics statement is available in the Methods section.

“This study was pre-registered and approved by the Heidelberg University Hospital Ethics Committee (S-271/2019).” (p.13, Methods section, revised manuscript)

*6. Please note that we cannot accept equations in an image format. They must be editable and we recommend either MathType or Microsoft Equation. In order to typeset properly, please also ensure that you format them in unicode font, which can be checked under 'Symbol' in Word.*

**Authors’ response:**

All equations are in editable format (Microsoft Equation, unicode font).

*ACKNOWLEDGMENTS: 1. Please remove the funding information from the Acknowledgments section of your manuscript file. The published version is pulled automatically from the “Financial Disclosure” field in our submission form and must not be duplicated. Please therefore take this opportunity to check that the information in this field is correct and completed in full.*

**Authors’ response:**

We have now removed funding information from the Acknowledgements section, as recommended. Additionally we have verified that all funding information in the online submission form is correct.

*2. Please remove the competing interest information from the Acknowledgements section of your manuscript file. The published version is pulled automatically from the “Competing Interests” field in our submission form and must not be duplicated. Please therefore take this opportunity to check that the information in that field is correct and completed in full.*

**Authors’ response:**

We have now removed the competing interest information from the Acknowledgements section of the manuscript. Additionally we have verified that all information in the submission form is correct.

*REFERENCES: 1. Please ensure your references are in the style of PLOS Medicine (ICMJE/Vancouver style). The reference style guide can be found here: http://journals.plos.org/plosmedicine/s/submission-guidelines#loc-references. You can also find a downloadable EndNote file in these guidelines to assist you. Please do a manual check as well, as the EndNote file does not catch everything. Please note that the references will be typeset as they appear so please ensure the format is consistent.*

**Authors’ response:**

We have used the provided reference style and have manually checked all references.

*2. Please put all reference citations in square brackets separated by either commas or dashes with no spaces, e.g. [1,2,3] or [1-3].*

**Authors’ response:**

We have used square brackets for all reference citations separated by commas or dashes with no spaces.

*3. Please make sure all references are cited in ascending numerical order in the text.*

**Authors’ response:**

We have verified all references in the text.

*SUPPORTING INFORMATION: 1. Please ensure your supporting information files are individually uploaded to the File Inventory as file type 'Supporting Information' and are not present in the manuscript file.*

**Authors’ response:**

We have uploaded all supporting information files as individual files to the File Inventory.

*2. Please upload your supporting information as individual files, and use the following naming format: S1 Text; S1 Fig; S1 Table, S1 Data, etc. Each file type should start as S1 and increase numerically: S1 Text, S2 Text, etc. Note that all relevant in-text citations and captions will need to be updated accordingly.*

**Authors’ response:**

We have used the recommended naming format.

*3. If you'd prefer to keep your supporting information in one file, please edit any citation of embedded figures and/or tables within your Supporting Information files and the main manuscript to use letters rather than numbers, e.g. “Table A in S1 Text” or "Fig B in S1 Appendix.".*

**Authors’ response:**

We have uploaded all supporting information files as individual files rather than one file.

*4. Please ensure that all Supporting Information files use the following format verbatim throughout the manuscript (including legends and all in-text citations): S1 Fig, S1 Table, S1 Text, S1 Data, etc. Each file type should start at 1 and increase numerically. Please note, however, that the file names themselves must use an underscore rather than a space, i.e. S1_Fig.tif, S1_Table.xlsx, S1_Text.doc, S1_Data.xlsx, S1_PRISMA_Checklist.docx, etc.*

**Authors’ response:**

We have verified that all Supporting Information files use the recommended format.

*5. Please ensure that each supporting information file has a legend at the end of the manuscript file, after the Reference list.*

**Authors’ response:**

We have now added legends at the end of the manuscript after the reference list.

*6. Please note that your submission cannot contain any references to generic "supporting information" files and instead must refer to specific files using our naming convention (S1 Fig, S1 Table, S1 Text, S1 Data, SI PRISMA Checklist, etc.)*

**Authors’ response:**

We have verified that our submission does not contain any references to generic supporting information.
